# Supplementary material for: Pretreatment with a γ-Secretase Inhibitor Prevents Tumor-like Overgrowth in Human iPSC-Derived Transplants for Spinal Cord Injury
Source: Stem Cell Reports. 2016 Sep 22;7(4):649–63. doi: 10.1016/j.stemcr.2016.08.015 (PMC5063571; doi:10.1016/j.stemcr.2016.08.015)
Supplement: Document S1. Supplemental Experimental Procedures, Figures S1–S5, and Tables S1 and S2 [file mmc1.pdf]

Stem Cell Reports, Volume 7

Supplemental Information

Pretreatment with a γ-Secretase Inhibitor Prevents Tumor-like Overgrowth in Human iPSC-Derived Transplants for Spinal Cord Injury

Toshiki Okubo, Akio Iwanami, Jun Kohyama, Go Itakura, Soya Kawabata, Yuichiro Nishiyama, Keiko Sugai, Masahiro Ozaki, Tsuyoshi Iida, Kohei Matsubayashi, Morio Matsumoto, Masaya Nakamura, and Hideyuki Okano

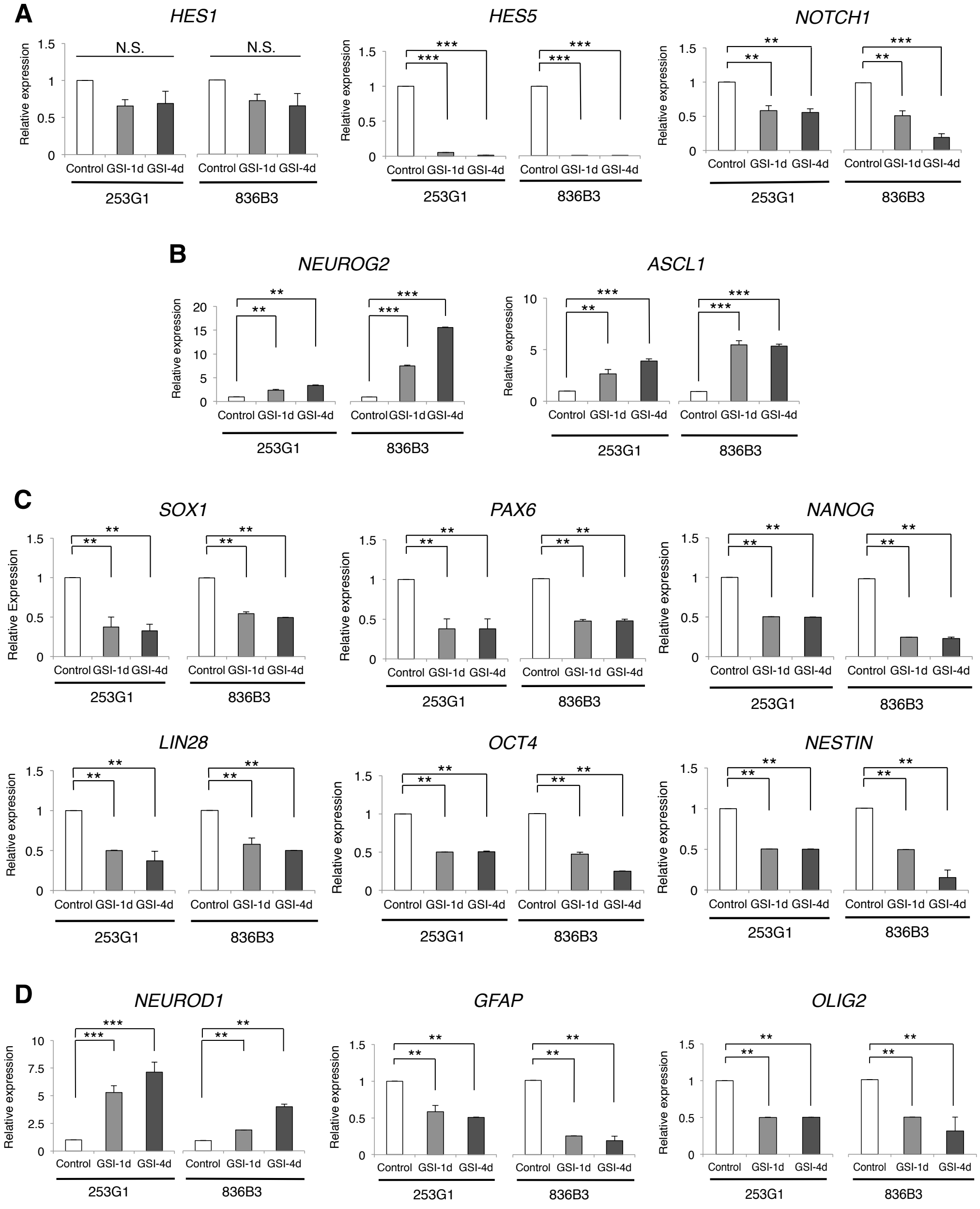

Figure S1. RT-PCR Analyses of hiPSC-NS/PCs Treated with GSI

(A-D) The gene expression of mRNA in the control, GSI-1d and GSI-4d groups were analyzed by RT-PCR for each cell line. Data, normalized to the reference GAPDH levels, were presented as expression levels relative to the control group (equal to 1) (n = 3 independent experiments).

$^{**}p < 0.01$, $^{***}p < 0.001$, N.S. = Non-significant, One-way ANOVA with the Tukey-Kramer test (A-D). The data are presented as the means ± SEM.

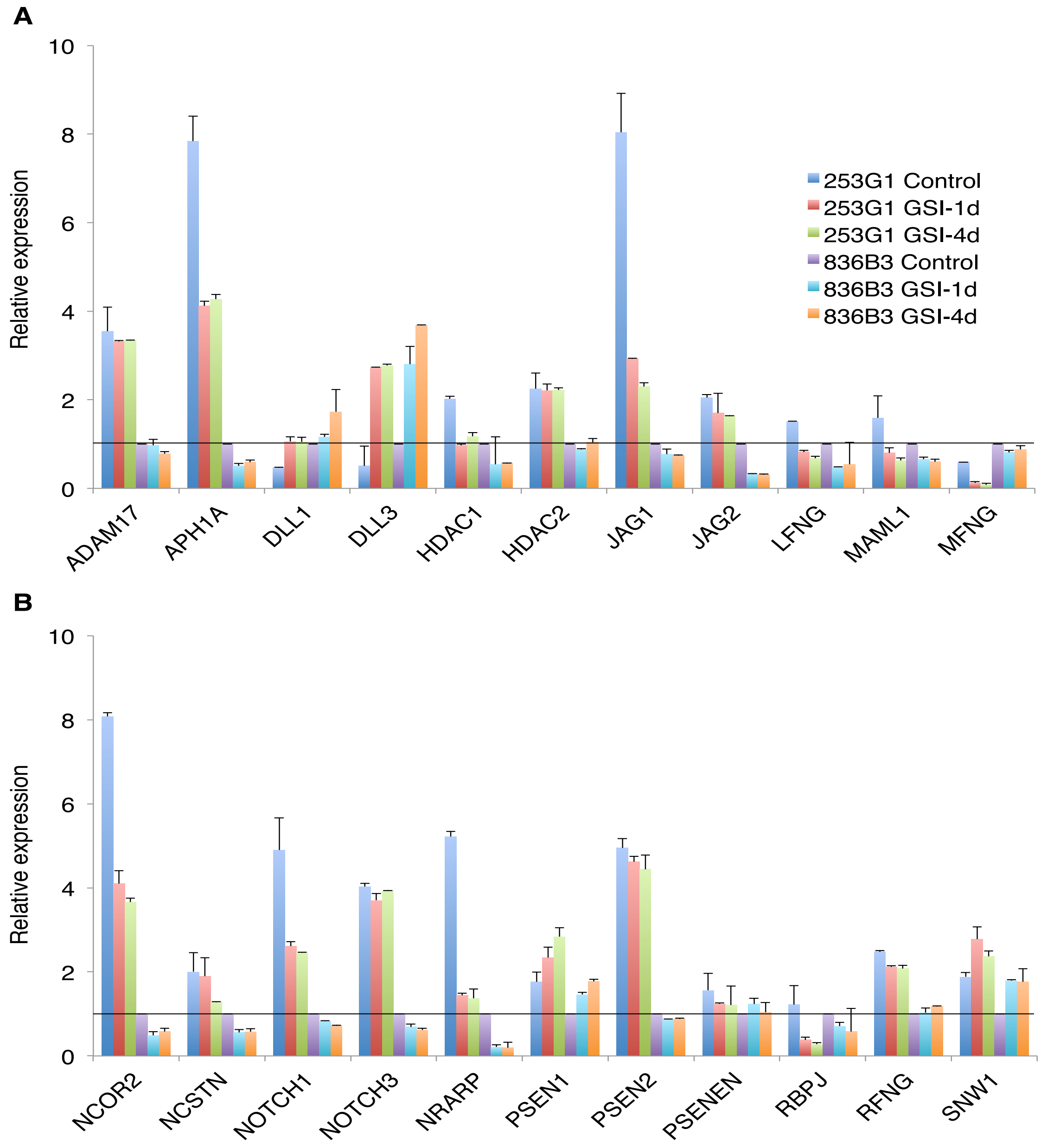

Figure S2. The Gene Expression Analyses of Human Notch Signaling Pathway in the hiPSC-NS/PCs Treated with or without GSI Using RT-PCR

(A, B) Quantitative RT-PCR analyses of known direct and indirect human Notch signal related genes for each group and cell lines. Data, normalized to the reference GAPDH levels, were presented as expression levels relative to the 836B3 hiPSC-NS/PCs control group (equal to 1) (n = 3 independent experiments).

One-way ANOVA with the Tukey-Kramer test (A, B). The data are presented as the means ± SEM.

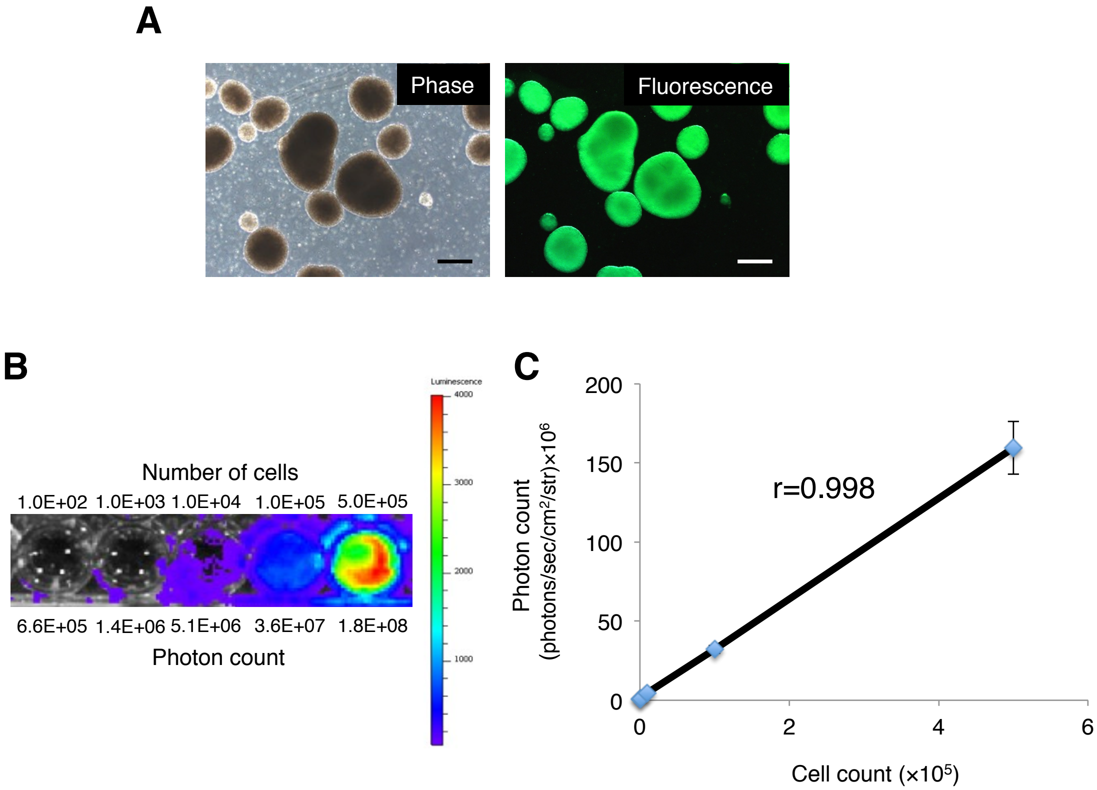

Figure S3. Detection of Bioluminescence and Fluorescence Signals in Lentivirally Transfected 253G1 hiPSC-NS/PCs *in vitro*

(A) Fluorescence image shows neurospheres of 253G1 hiPSC-NS/PCs expressing the fluorescent protein cp156-Venus, which is originally modified from GFP.

(B) Bioluminescence imaging was used to detect bioluminescence signals in various numbers of 253G1 hiPSC-NS/PCs (0, 1×10^2, 1×10^3, 1×10^4, 1×10^5, and 5×10^5 cells per well).

(C) BLI significantly find out a direct linear correlation between cell numbers and photon counts in vitro (n = 5 independent experiments).

The data are presented as the means ± SEM. Scale bars, 200 µm (A).

BLI; bioluminescence imaging

A

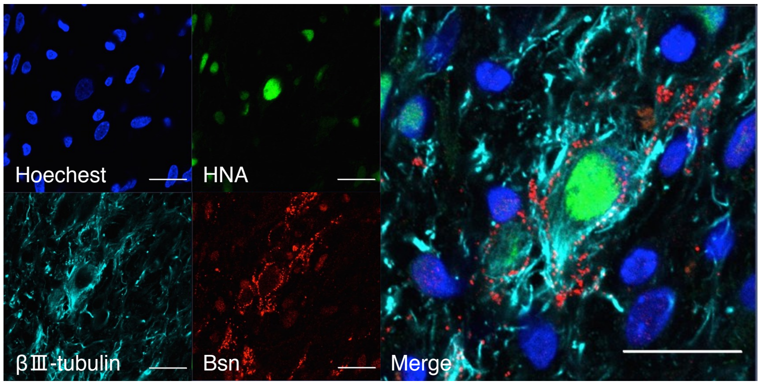

B

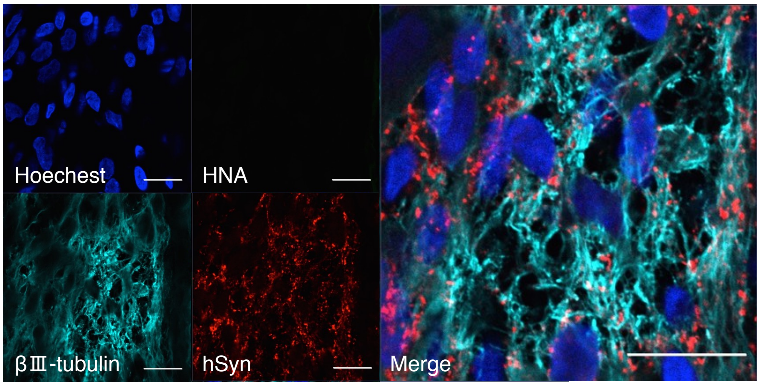

Figure S4. Transplanted hiPSC-NS/PCs Contribute to Synapse Formation between Transplanted Cell-Derived Neurons and Host Mouse Neurons

(A) Representative images of immunohistochemistry show the staining for HNA, βⅢ tubulin, and the mouse presynaptic marker Bsn. βⅢ tubulin$^+$/HNA$^+$ transplanted cell-derived neurons were observed in contact with Bsn$^+$ cells.

(B) Representative images of immunohistochemistry show the staining for HNA, βⅢ tubulin, and the human-specific presynaptic marker hSyn. hSyn$^+$ boutons were apposed to βⅢ tubulin$^+$/HNA$^-$ host mouse neurons.

Scale bars, 20μm (A and B).

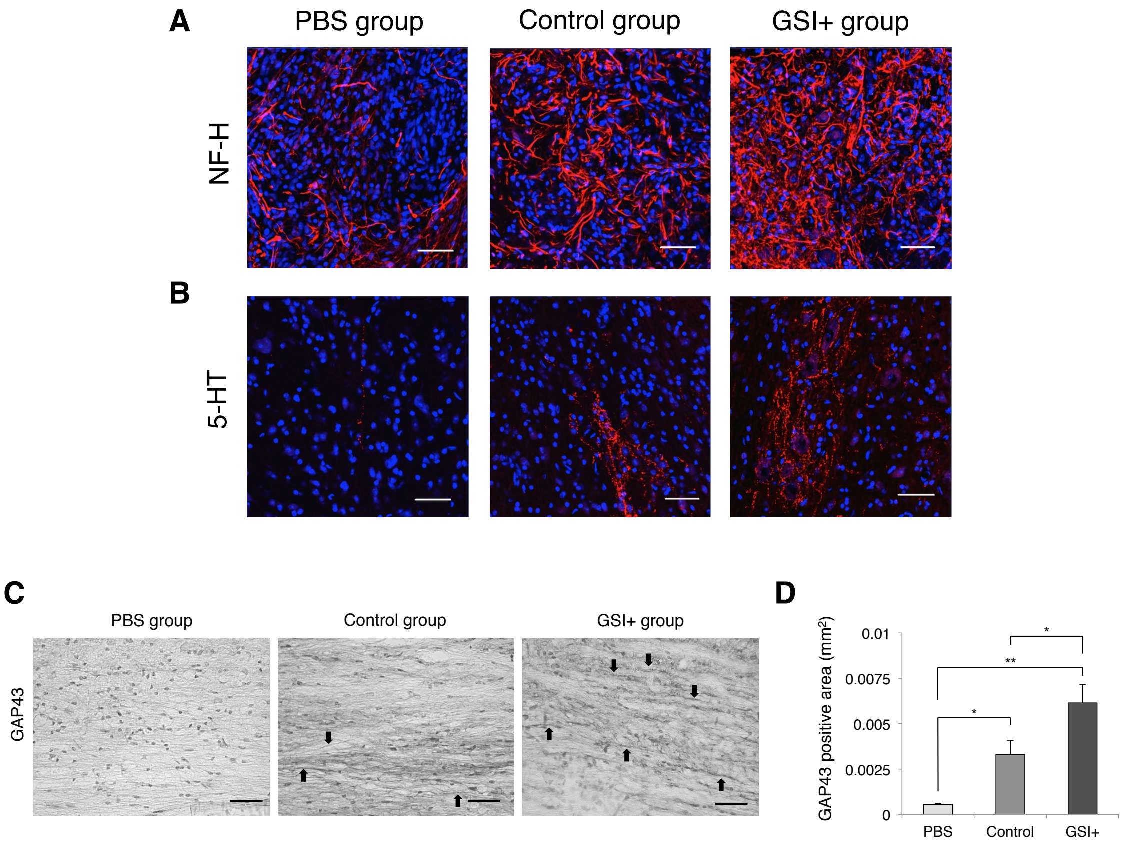

Figure S5. Transplanted hiPSC-NS/PCs with GSI Treatment Enhanced Axonal Regrowth after SCI.

(A) Representative images of the immunohistochemical staining for NF-H at the lesion epicenter, and (B) 5-HT at the lumbar intumescence, show the results for each group.

(C) Representative images of midsagittal sections stained for GAP43 in the ventral region 1 mm caudal to the lesion epicenter. Black arrows indicate the GAP43^{+} fibers

(D) Quantitative analyses of the GAP43+ area in midsagittal sections.

$^{*}p < 0.05$, $^{**}p < 0.01$ according to a one-way ANOVA with the Tukey-Kramer test (D). The data are presented as the means ± SEM. Scale bars, 50μm (A-C).

Table S1. Gene Ontology analysis of human genes upregulated and downregulated decreased in 253G1 hiPS-NS/PCs after pretreated with GSI-1d and GSI-4d

GO:Accession	GO Term	Corrected p-value
GO:0030182	neuron differentiation	1.38E-17
GO:0048666	neuron development	7.31E-09
GO:0007409	axonogenesis	8.90E-09
GO:0048812	neuron projection morphogenesis	9.17E-09
GO:0031175	neuron projection development	2.22E-07
GO:0007411	axon guidance	6.05E-07
GO:0051726	regulation of cell cycle	6.58E-03
GO:0021700	developmental maturation	6.80E-03
GO:0043066	negative regulation of apoptosis	1.17E-02
GO:0043069	negative regulation of programmed cell death	1.31E-02
GO:0060548	negative regulation of cell death	1.35E-02
GO:0010717	regulation of epithelial to mesenchymal transition	1.56E-02
GO:0042981	regulation of apoptosis	1.85E-02
GO:0010941	regulation of cell death	2.17E-02
GO:0008219	cell death	4.45E-02

Table S2. Gene Ontology analysis of human genes upregulated and downregulated decreased in 836B3 hiPS-NS/PCs after pretreatment with GSI-1d and GSI-4d

GO:Accession	GO Term	Corrected p-value
GO:0030182	neuron differentiation	3.65E-07
GO:0048666	neuron development	1.31E-04
GO:0048663	neuron fate commitment	2.08E-04
GO:0021700	developmental maturation	7.15E-04
GO:0048469	cell maturation	1.89E-03
GO:0045597	positive regulation of cell differentiation	5.47E-03
GO:0051726	regulation of cell cycle	8.66E-03
GO:0001764	neuron migration	1.02E-02
GO:0010720	positive regulation of cell development	1.25E-02
GO:0021515	cell differentiation in spinal cord	1.31E-02
GO:0021954	central nervous system neuron development	2.11E-02
GO:0021510	spinal cord development	3.01E-02
GO:0021953	central nervous system neuron differentiation	3.20E-02
GO:0031175	neuron projection development	3.42E-02
GO:0007409	axonogenesis	4.69E-02

Supplemental Experimental Procedures

Cell Culture, NS/PCs Derived from hiPSC-NS/PCs Formation Assay, Neuronal Differentiation Analyses and Lentiviral Transduction

Three human induced pluripotent stem cells (hiPSCs) lines; clone 253G1 (Nakagawa et al., 2008) generated following retroviral transfection of 3-factors (Oct3/4, Sox2 and Klf4) and clone 836B3 (Maekawa et al., 2011; Okita et al., 2011) reprogrammed with episomal plasmid vectors containing 6-factors (Oct3/4, Sox2, Klf4, L-Myc, LIN28 and Glis1), a potentially tumor-like overgrowth, and normal clone 201B7 (Miura et al., 2009) generated following retroviral transfection of 4-factors (Oct3/4, Sox2, Klf4 and c-myc) were cultured for 12 days on adhesion culture with mouse embryonic fibroblast (MEF), and formed into embryo bodies in floating culture for 30 days. Aggregate cells were differentiated into NS/PCs derived from hiPSC-NS/PC formation using various factors during each day of the incubation period. In the analyses of neuronal differentiation, hiPSC-NS/PCs were plated onto poly-L-ornithine/fibronectin-coated 48-well chamber slides (Costar 3548; Corning, NY, USA) at a density of 1×10^5 cells/ml and cultured in medium without growth factors at 37 °C in 5% CO_2 and 95% air for 14 days. Differentiated cells were fixed with 4% paraformaldehyde (PFA) in 0.1 M phosphate buffered saline (PBS) and stained with the following primary antibodies for immunocytochemistry: anti-Ki67 (rabbit IgG, 1:500, Leica Biosystems, Wetzlar, Germany), anti-human-specific Nestin protein (mouse IgG1, 1:500, Merck Millipore, Billerica, Massachusetts, USA), anti-βⅢ-tubulin (mouse IgG2b, 1:500, Sigma-Aldrich, St. Louis, MO, USA), anti-glial fibrillary acidic protein (GFAP, rabbit IgG, 1:200, Dako, Carpinteria, CA, USA), and anti-Cyclicnucleotide phosphodiesterase (CNPase, mouse IgG1, 1:4000, Sigma-Aldrich, St. Louis, MO, USA). Nuclei were stained with Hoechst 33258 (10 μg/ml, Sigma-Aldrich, St. Louis, MO, USA). All *in vitro* images were obtained using a confocal laser scanning microscopy (LSM 700; Carl Zeiss, Jena, Germany / In Cell Analyzer 6000; GE health care UK Ltd, Little Chalfont, Buckinghamshire, UK).

Flow Cytometric Analyses

EdU was added to the culture medium at a final concentration of 10 μM and mixed well for one hour at 37 ℃. Subsequently, cells were washed with 1% Bovine serum albumin (BSA) in PBS, removed the supernatant, fixed for 15 minutes, incubated with Click-iT Plus reaction cocktail (anti-EdU antibodies included) for 30 minutes, and stained FxCycle™ Violet (Life technologies, Carlsbad, CA, USA). Flow cytometry and data

analyses were performed on a fluorescence-activated cell sorting (FACS) Calibur instrument (BD Biosciences, San Jose, CA, USA).

Micro Electrode Array

hiPSC-NS/PCs were dissociated into single cells and also plated in culture medium on the human laminin-coated MEA plates (*n* = 3, 12 well plate, Axion Biosystems, Atlanta, GA, USA). The data were acquired using a sampling rate of 12.5 kHz and filtered using a 200–3000 Hz Butterworth band-pass filter. A detection threshold was set to +6.0×SD of the baseline electrode noise. The spike count files generated from recordings were used to calculate the number of active electrodes, which were defined as an electrode having an average of more than five spikes/min in each well. The first three minutes of each data file was removed in order to allow the activity to stabilize in the Maestro and 10-15 min of activity was recorded. MEA data analyses were performed by NeuroExplorer (Nex Technologies, Madison, AL, USA) software, which normalized the baseline neuronal activity and plotted as a graph for each condition.

DNA Microarray Analyses

Total RNA was extracted from the hiPSC-NS/PCs using an RNeasy Micro Kit (Qiagen Inc., Hilden, Germany) according to the manufacturer’s instructions. For the DNA microarray analyses, the RNA quality was assessed using a 2100 Bioanalyzer (Agilent Technologies Inc., Santa Clara, CA, USA), and 100 ng of total RNA was reverse transcribed, biotin labeled, and hybridized to a Human HT-12v4.0 Expression BeadChip (Illumina Inc., San Diego, CA, USA). The array was then washed and stained in a Fluidics Station 450, according to the manufacturer’s instructions. The microarrays were scanned using an iScan system (Illumina Inc., San Diego, CA, USA), and the raw image files were converted to normalized signal intensity values using the GenomStudio algorithm (Illumina Inc., San Diego, CA, USA). The results were normalized and narrowed down by the cut-off values for the expression levels (>50) and by the fold changes (>2.0, versus the signal of the ‘Control group’). GO analyses were performed using gene lists from the overlapping area in the Venn diagram of each hiPSC-NS/PC. P values were calculated using Fisher’s exact test.

RT-PCR

Total RNA was extracted from hiPSC-NS/PCs by using RNeasy Micro Kit (Qiagen Inc., Hilgen, Germany), and reverse transcribed with the ReverTra Ace qPCR RT master mix (TOYOBO co., Ltd. Life science

department, Osaka, Japan). Quantitative polymerase chain reactions (QT-PCR) were performed using Step One Plus^{TM} (Applied Biosystems, Foster city, CA, USA), according to the manufacturer's instructions. The expression levels of each gene were normalized to that of GAPDH using the ΔΔCT method. We chose primers used for the reactions from TaqMan gene expression assays and TaqMan gene expression 96-well fast plates (Life technologies, Carlsbad, CA, USA).

Bioluminescence Imaging

A Xenogen-IVIS spectrum cooled, charge coupled device (CCD) optical macroscopic imaging system (Caliper Life-Science, Hopkinton, MA, USA) was used for BLI to evaluate the survival of the hiPSC-NS/PCs grafted as described in the literature (Itakura et al., 2014; Okada et al., 2005; Takahashi et al., 2011). The signal intensity of hiPSC-NS/PCs was assessed *in vitro* using cells plated at various cell numbers (approximate range 1×10^2 to 5×10^5 cells/well), and BLIs were performed immediately after adding D-luciferin (150 µg/ml; Promega, Madison, WI, USA, n=3). The integration time was fixed at five minutes for each image. Imaging was performed *in vivo* five minutes after via i.p. injection of D-luciferin (0.3 mg/g body weight) with the field-of-view set at 13.2 cm, as the photon count was most stable during this period. The intensity peaked between 10 and 30 minutes. All images were measured with Living Image software, and the optical signal intensity was expressed as photon count in units of photons/sec/cm^2/str. Each result was displayed as a pseudo-colored photon count image superimposed on a gray-scale anatomic image. A region of interest was defined in the area cell-implanted, and all values at the same region of interest were elucidated to quantify the light measured. The survival and growth of cells transplanted in the mouse spinal cord was measured by BLI for IVIS system every week until 89 days after transplantation.

SCI Animal Model and hiPSC-NS/PCs Transplantation

Adult female NOD-SCID mice (8-weeks-old, 20-22 g; Clea, Tokyo, Japan) were anesthetized with intra-peritoneal (i.p.) injection of ketamine (100 mg/kg) and xylazine (10 mg/kg). After laminectomy at the level of 10th thoracic spinal vertebra, the dorsal surface of the dura mater was exposed, and contusive SCI was induced using an IH impactor (a force defined impact (60 kdyn) with a stainless steel-tipped impactor; Precision Systems and Instrumentation, Lexington, KY, USA) as described previously (Scheff et al., 2003). Donor cells were prepared *in vitro* from 253G1 and 201B7 hiPSC-NS/PCs, which were cultured with GSI for one day in 'GSI+ group' and without GSI in 'control group' before cell transplantation. Nine days after SCI,

hiPSC-NS/PCs pretreating with or without GSI (5×10^5 cells/2 μl) were transplanted into the lesion epicenter of each mouse ('control group' and 'GSI+ group', n=10 each) with a glass micropipette at a rate of 1 μl/min using a Hamilton syringe (25 μl) and a stereotaxic micro injector (KDS 310; Muromachi Kikai Co. Ltd., Tokyo, Japan). An equal volume of PBS was injected instead of 'PBS group' mice.

Histological analyses

Animals were anesthetized and transcardially euthanized with 0.1 M PBS containing 4% PFA at 89 days after transplantation. Spinal cords were removed, post-fixed overnight in 4% PFA, soaked overnight in 10% sucrose, followed by 30% sucrose, embedded in Optimal Cutting Temperature (O.C.T) compound (Sakura Finetechnical Co., Ltd., Tokyo, Japan), frozen, and sectioned in the sagittal plane at 16 μm thickness on a cryostat (CM3050S, Leica Microsystems, Wetzlar, Germany). Spinal cord sections were histologically evaluated by staining with HE and immunohistochemistry (IHC). Especially, tissue sections were applied and stained with the following primary antibodies for IHC: anti-pan-ELAVL (Hu) (human IgG, 1:1000, a gift from Dr. Robert Darnell, The Rockefeller University, New York, NY, USA), anti-GFAP (rabbit IgG, 1:200, Dako, Carpinteria, CA, USA, Z0334), anti-APC CC-1 (mouse IgG, 1:300; Abcam, Cambridge, UK, ab16794), anti-human-specific Nestin protein (rabbit IgG, 1:300; described previously (Kanemura et al., 2002; Nakamura et al., 2003)), anti-Ki67 (rabbit IgG, 1:200; Leica Biosystems, Wetzlar, Germany), anti-human nuclear antigen (anti-HNA, mouse IgG, 1:200; Chemicon, Temecula, CA, USA, MAB1281), anti-Notch1 (rabbit IgG, 1:100; Abcam, Cambridge, UK, ab65297), anti-activated Notch1 (rabbit IgG, 1:100; Abcam, Cambridge, UK, ab8925), anti-Snail (rabbit IgG, 1:100; Abcam, Cambridge, UK, ab180714), anti-βⅢ-tubulin (Tuj1; mouse IgG, 1:300; Sigma-Aldrich, St. Louis, MO, USA, T8660), anti-Bsn (mouse IgG, 1:200; Stressgen, ADI-VAM-PS003), anti-hSyn (Mouse IgG, 1:200; Chemicon, Temecula, CA, USA, MAB332), anti-neurofilament 200 kDa (NF-H, mouse IgG, 1:200; Merck Millipore, Billerica, Massachusetts, USA, MAB5266), anti-5-hydroxytrypamine (5HT, goat IgG, 1:200; Immunostar), anti-growth associated protein 43 (GAP43, mouse IgG, 1:1000; Chemicon, Temecula, CA, USA). For immunohistochemistry with anti-GAP43, we used a biotinylated secondary antibody (Jackson Immunoresearch Laboratory, West Grove, PA, USA), after exposure to 0.3% H_2O_2 for 60 min at room temperature to inactivate the endogenous peroxidase. The signals were then en- hanced with the Vectastain ABC kit (Vector Laboratories, Burlingame, CA, USA). Nuclei were stained with Hoechst 33258 (10 μg/ml, Sigma-Aldrich, St. Louis, MO, USA). Samples were elucidated on a fluorescence microscope (BZ 9000; Keyence Co., Osaka, Japan) or a confocal laser-scanning

microscope (LSM 700, Carl Zeiss, Jena, Germany). The numbers of marker-positive cells, such as HNA-, Ki67-, Nestin-, pan-ELAVL (Hu)-, GFAP-, APC-, Notch1-, Activated Notch1-, Snail- positive cells, were counted in each section (n = five per group).

Behavioral Analyses

Two investigators blinded to identify the experimental mice performed this assessment. Motor coordination was evaluated using a rotating rod apparatus (Rota-rod, Muromachikikai Co., Ltd.), which consisted of a plastic rod (three cm diameter, eight cm length) with a gridded surface flanked by two large discs (40 cm diameter). Each mouse was placed on the rod while it rotated at 20 rpm for two minutes sessions at 89 days after transplantation. Five trials were conducted, and the maximum number of seconds the mouse stayed on the rod was recorded. Gait analyses were performed using the DigiGait system (Mouse Specifics, Quincy, MA, USA, n=five per group). Each mouse demonstrated weight-supported hindlimb stepping at 89 days after transplantation. Stride length was determined on a treadmill set to a speed of seven cm/s.

Supplemental References

Maekawa, M., Yamaguchi, K., Nakamura, T., Shibukawa, R., Kodanaka, I., Ichisaka, T., Kawamura, Y., Mochizuki, H., Goshima, N., and Yamanaka, S. (2011). Direct reprogramming of somatic cells is promoted by maternal transcription factor Glis1. Nature *474*, 225-229

Nakagawa, M., Koyanagi, M., Tanabe, K., Takahashi, K., Ichisaka, T., Aoi, T., Okita, K., Mochiduki, Y., Takizawa, N., and Yamanaka, S. (2008). Generation of induced pluripotent stem cells without Myc from mouse and human fibroblasts. Nat. Biotechnol. *26(1)*, 101-106

Okita, K., Matsumura, Y., Sato, Y., Okada, A., Morizane, A., Okamoto, S., Hong, H., Nakagawa, M., Tanabe, K., Tezuka, K., et al. (2011). A more efficient method to generate integration-free human iPS cells. Nature Methods *8*, 409-412
