# Supplementary material for: Pretreatment with a γ-Secretase Inhibitor Prevents Tumor-like Overgrowth in Human iPSC-Derived Transplants for Spinal Cord Injury
Source: Stem Cell Reports. 2016 Sep 22;7(4):649–63. doi: 10.1016/j.stemcr.2016.08.015 (PMC5063571; doi:10.1016/j.stemcr.2016.08.015)
Supplement: Document S2. Article plus Supplemental Information [file mmc2.pdf]

Stem Cell Reports
Article
OPEN ACCESS

Pretreatment with a γ-Secretase Inhibitor Prevents Tumor-like Overgrowth in Human iPSC-Derived Transplants for Spinal Cord Injury

Toshiki Okubo,[1,2] Akio Iwanami,[1] Jun Kohyama,[2] Go Itakura,[2] Soya Kawabata,[1] Yuichiro Nishiyama,[1] Keiko Sugai,[1] Masahiro Ozaki,[1,2] Tsuyoshi Iida,[1,2] Kohei Matsubayashi,[1,2] Morio Matsumoto,[1] Masaya Nakamura,[1,*] and Hideyuki Okano[2,*]
[1]Department of Orthopaedic Surgery
[2]Department of Physiology
Keio University School of Medicine, 35 Shinanomachi, Shinjuku-ku, Tokyo 160-8582, Japan
*Correspondence: masa@a8.keio.jp (M.N.), hidokano@a2.keio.jp (H.O.)
http://dx.doi.org/10.1016/j.stemcr.2016.08.015

SUMMARY

Neural stem/progenitor cells (NS/PCs) derived from human induced pluripotent stem cells (hiPSCs) are considered to be a promising cell source for cell-based interventions that target CNS disorders. We previously reported that transplanting certain hiPSC-NS/PCs in the spinal cord results in tumor-like overgrowth of hiPSC-NS/PCs and subsequent deterioration of motor function. Remnant immature cells should be removed or induced into more mature cell types to avoid adverse effects of hiPSC-NS/PC transplantation. Because Notch signaling plays a role in maintaining NS/PCs, we evaluated the effects of γ-secretase inhibitor (GSI) and found that pretreating hiPSC-NS/PCs with GSI promoted neuronal differentiation and maturation in vitro, and GSI pretreatment also reduced the overgrowth of transplanted hiPSC-NS/PCs and inhibited the deterioration of motor function in vivo. These results indicate that pretreatment with hiPSC-NS/PCs decreases the proliferative capacity of transplanted hiPSC-NS/PCs, triggers neuronal commitment, and improves the safety of hiPSC-based approaches in regenerative medicine.

INTRODUCTION

Embryonic stem cells and induced pluripotent stem cells (iPSCs) can differentiate into neural stem/progenitor cells (NS/PCs), which can subsequently be induced in vitro to differentiate into three neural lineages: neurons, astrocytes, and oligodendrocytes (Falk et al., 2012; Miura et al., 2009; Okada et al., 2004). Furthermore, accumulating evidence suggests that NS/PCs represent a promising cell source for regenerative medicine targeting CNS disorders (Cummings et al., 2005; Hofstetter et al., 2005; Iwanami et al., 2005; Kumagai et al., 2009; Nori et al., 2011; Okada et al., 2005, 2008; Ogawa et al., 2002; Salazar et al., 2010; Yasuda et al., 2011). Our previous reports have shown that transplantation of NS/PCs derived from human induced pluripotent stem cells (hiPSC-NS/PCs) promotes motor function recovery in non-obese diabetic-severe combined immune-deficient (NOD-SCID) mice and non-human primates with spinal cord injury (SCI) (Fujimoto et al., 2012; Kobayashi et al., 2012; Nori et al., 2011; Okano et al., 2013; Tsuji et al., 2010). However, transplanting certain hiPSC-NS/PCs, such as clone 253G1 (generated through a process of retroviral transfection), results in tumor-like overgrowth and deterioration of motor function during long-term observations (Nori et al., 2015), and transplanting clone 836B3 (episomal plasmid vectors) in an SCI animal model yielded similar results during long-term observations (our unpublished data). Moreover, these tumors consisted of undifferentiated human-specific Nestin$^+$ cells.

The safety of measures for preventing tumor-like overgrowth is of great importance in clinical applications of iPSC-based transplantation therapy for SCI. Remnant immature NS/PCs must be removed or induced to differentiate into more mature cell types, which may avoid tumor-like overgrowth following transplantation. Notch signaling controls the induction of NS/PCs, and inhibition of this signaling with a γ-secretase inhibitor (GSI) induces the NS/PCs to develop into a more mature state with limited proliferation in vitro (Crawford and Roelink, 2007; Nelson et al., 2007). Treatment of iPSC-derived dopaminergic progenitor cells with GSIs prior to transplantation into the normal mid-striatum is known to control the growth of a potentially proliferative cell population in vivo (Ogura et al., 2013).

The purpose of the present study was to elucidate the effects of a GSI on the proliferation and differentiation of tumorigenic hiPSC-NS/PCs in vitro, assess the effects of GSI pretreatment on the hiPSC-NS/PCs in vivo, and determine whether animal models of SCI exhibit recovered motor functions and an absence of tumor-like overgrowth following transplantation of the pretreated cells.

RESULTS

Treatment with the GSI Suppressed the Proliferation of hiPSC-NS/PCs

We performed differentiation and proliferation assays using hiPSC-NS/PCs in vitro. After treating the cells with

© 2016 The Author(s).
This is an open access article under the CC BY license (http://creativecommons.org/licenses/by/4.0/).

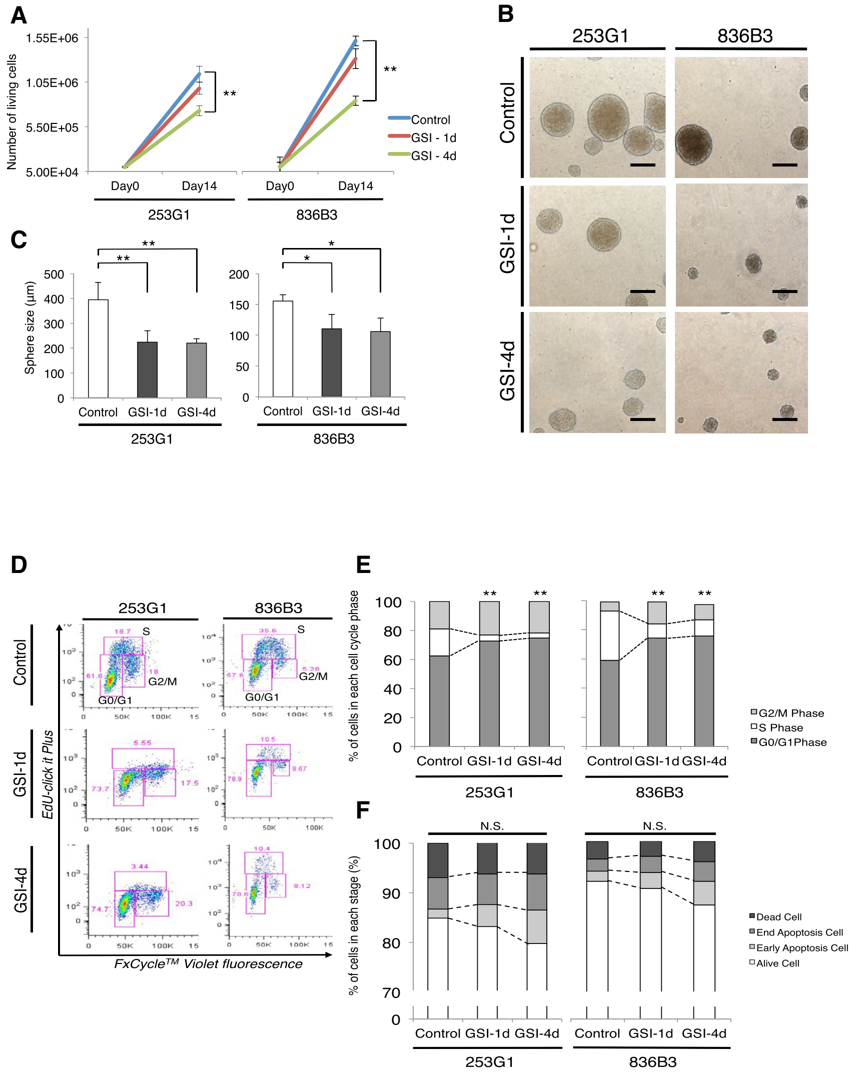

Figure 1. Proliferation of hiPSC-NS/PCs Treated with or without GSI

(A) Number of living cells counted in ten separate fields using a microscope and compared among each group (n = 5 independent experiments).

(B) Micrographs showing representative hiPSC-NS/PC aggregates for each group and cell line. Scale bars, 200 μm.

(C) Size of hiPSC-NS/PCs measured in ten separate fields using a microscope and compared among each group (n = 5 independent experiments).

(D) Cell-cycle analyses of hiPSC-NS/PCs performed using flow cytometry. Representative dot plots are shown for each group.

(E) Histograms show the relative distribution of hiPSC-NS/PCs across the different cell cycle phases under self-renewing conditions (n = 5 independent experiments).

(F) Analyses of hiPSC-NS/PC apoptosis performed using the annexin V/7-AAD apoptosis assay. The histograms show the relative distribution of hiPSC-NS/PCs across the different phases (n = 5 independent experiments).

$^{*}p < 0.05$, $^{**}p < 0.01$, and not significant (N.S.) according to one-way ANOVA with the Tukey-Kramer test. Data are presented as means ± SEM.

or without GSI, aggregated hiPSC-NS/PCs were dissociated into single cells and the living cells were counted. In the GSI-4d group (hiPSC-NS/PCs cultured in vitro with GSI for 4 days), the number of living cells was significantly decreased compared with that of the other groups (253G1: control 1.14×10^6 cells, GSI-1d [hiPSC-NS/PCs cultured in vitro with GSI for 1 day] 9.80×10^5 cells, GSI-4d 7.28×10^5 cells; 836B3: control 1.51×10^6 cells, GSI-1d 1.31×10^6 cells, GSI-4d, 8.42×10^5 cells; Figure 1A). Next, the size of the sphere was measured by microscopy after treatment with or without GSI. In the control group, the size of the sphere was significantly increased compared with that of both GSI groups (253G1: control 394.7 ± 69.5 μm, GSI-1d 224.1 ± 46.1 μm, GSI-4d 220.4 ± 17.3 μm; 836B3: control 155.2 ± 10.7 μm, GSI-1d 110.4 ± 23.6 μm, GSI-4d 105.9 ± 21.8 μm; Figures 2B and 2C).

In the cell-cycle analyses, representative dot plots of the flow cytometry data revealed a reduced S-phase population among the GSI-treated hiPSC-NS/PCs (Figure 1D). Compared with the control group, the proportion of cells in G_0/G_1 phase was significantly increased (253G1: control 62.6% ± 2.7%, GSI-1d 72.8% ± 1.1%, GSI-4d 74.8% ± 1.8%; 836B3: control 58.9% ± 1.3%, GSI-1d 74.4% ± 2.3%, GSI-4d 75.7% ± 2.9%). The proportion of cells in the S phase was significantly decreased in both GSI groups (253G1: control 18.6% ± 0.8%, GSI-1d 4.1% ± 0.2%, GSI-4d 3.6% ± 0.1%; 836B3: control 33.8% ± 0.9%, GSI-1d 9.6% ± 0.6%, GSI-4d 11.2% ± 1.2%; Figure 1E). According to the annexin V/7-AAD apoptosis assay, the proportion of early apoptotic cells was slightly increased in both GSI groups compared with the control group, although significant differences were not observed among these groups (253G1: control 2.01% ± 0.2%,

GSI-1d 4.1% ± 0.3%, GSI-4d 5.5% ± 0.4%; 836B3: control 2.0% ± 0.5%, GSI-1d 3.1% ± 0.3%, GSI-4d 4.7% ± 0.5%). These results suggest that GSI treatment could induce the differentiation of undifferentiated hiPSC-NS/PCs.

GSI Induced Differentiation into More Mature Neuronal Cell Types with Limited Cell Proliferation

Next, we assessed the effects of GSI on the neuronal differentiation and maturation of hiPSC-NS/PCs. In both GSI groups, the proportion of Ki-67$^+$ (a cell proliferation marker) and Nestin$^+$ (a neural stem cell marker) cells was also significantly decreased compared with that of the control group (Ki-67$^+$ cells: 253G1—control 45.2% ± 1.4%, GSI-1d 30.7% ± 2.2%, GSI-4d 28.1% ± 0.8%; 836B3—control 34.0% ± 2.7%, GSI-1d 16.7% ± 1.9%, GSI-4d 13.0% ± 1.7%; Nestin$^+$ cells: 253G1—control 60.8% ± 1.9%, GSI-1d 36.6% ± 1.2%, GSI-4d 30.9% ± 1.4%; 836B3—control 43.6% ± 2.1%, GSI-1d 34.1% ± 0.5%, GSI-4d 23.6% ± 1.1%; Figures 2A–2D), whereas the proportion of βIII-tubulin$^+$ neurons was significantly increased (253G1: control 70.9% ± 2.3%, GSI-1d 94.7% ± 3.1%, GSI-4d 94.3% ± 2.4%; 836B3: control 47.6% ± 4.8%, GSI-1d 86.0% ± 4.9%, GSI-4d 86.7% ± 5.0%). The proportion of GFAP$^+$ astrocytes and CNPase$^+$ (2′,3′-cyclic-nucleotide 3′-phosphodiesterase) oligodendrocytes was not significantly different among the three groups (Figures 2E–2H). Microelectrode array (MEA) analyses indicated that neuronal maturation was significantly enhanced in both GSI groups compared with the control group (Figure 2J).

Gene Expression Profiles of hiPSC-NS/PCs after the GSI Treatment

DNA microarray analyses were performed on the hiPSC-NS/PCs that were treated with or without GSI to evaluate the gene expression profiles of the cells. Following the GSI treatment, 61 genes were downregulated (downregulation: 97 genes in the GSI-1d group, 274 genes in the GSI-4d group; Figure 3A) and 53 genes were upregulated (upregulation: 58 genes in the GSI-1d group, 147 genes in the GSI-4d group; Figure 3B) in 253G1 hiPSC-NS/PCs. In 836B3 hiPSC-NS/PCs, 26 genes were downregulated (downregulation: 49 genes in the GSI-1d group, 63 genes in the GSI-4d group; Figure 3C) and seven genes were upregulated (upregulation: eight genes in the GSI-1d group, 77 genes in the GSI-4d group; Figure 3D). Functional association analyses showed that the expression of cell-cycle-related genes and more mature neuronal marker-related genes increased by >2-fold in both GSI groups compared with the control group. Significant increases in the levels of p53-dependent genes, such as *CDKN1C* and *GADD45G*, and more neuronal maturation-dependent genes, such as *NEUROG1*, *NEUROD1*, and *ASCL1*, were detected (Figures 3E and 3F).

Gene Ontology (GO) analyses identified a number of neuronal differentiation- and maturation-associated terms, such as "neuron differentiation," "neuron development," "developmental maturation," "regulation of cell cycle," and "neuron projection development," in the GSI-treated hiPSC-NS/PCs (Tables S1 and S2).

We performed RT-PCR to validate these findings, which showed that the expression of representative Notch signaling target genes, such as *HES5* and *NOTCH1*, was nearly abolished by pretreatment with GSI (Figure S1A). In addition, the expression of early neural marker genes, such as *SOX2* and *PAX6*, was also downregulated in both GSI groups. The expression of mature neuronal marker genes, such as *NEUROG2*, *ASCL1*, and *NEUROD1* (but not glial markers, such as *GFAP* and *OLIG2*), was upregulated, as expected, given the downregulated expression of pluripotency/self-renewal markers, such as *NANOG*, *LIN28*, *OCT4*, and *NESTIN* (Figures S1B–S1D).

Next, we confirmed the role of Notch signaling in the specification of hiPSC-NS/PCs by comparing the qRT-PCR analyses for each cell line after treatment with or without GSI. The data were presented as expression levels relative to the 836B3 hiPSC-NS/PCs. The expression of Notch signaling-related genes, such as *JAGGED1*, *NOTCH1*, and *PSEN1*, was significantly upregulated in the 253G1 hiPSC-NS/PCs, which seemed to indicate that Notch signaling was increased in the 253G1 hiPSC-NS/PCs compared with the 836B3 hiPSC-NS/PCs. Under these activation conditions, the expression of the *HES5*, *HEYL*, *HSPB1*, *ID2*, *ID4*, *NESTIN*, *OCT3/4*, *VCAM1*, and *NANOG* genes were particularly downregulated in the GSI-treated groups compared with the control group. Additionally for each cell line, the GSI treatment resulted in a decrease in the expression of the target genes of Notch signaling, such as *JAGGED1* and *NRARP* (Figures S2A and S2B).

Previous transcriptome analyses by our group revealed the altered expression of genes involved in epithelial-mesenchymal transition (EMT), which plays roles in tumor invasion and progression, following the transplantation of 253G1 hiPSC-NS/PCs (Nori et al., 2015). The expression of EMT-related genes, such as *SNAIL1*, *SNAIL2*, *TWIST1*, and *TWIST2*, was downregulated in the GSI-treated group compared with the control group (Figure 3G).

Long-Term Observations after Transplantation of Tumorigenesis-/Overgrowth-Prone hiPSC-NS/PCs that Had Been Pretreated with GSI

Next, we examined the long-term effects of GSI pretreatment on hiPSC-NS/PCs that had been transplanted into SCI model mice in vivo and focused on the potency of the GSI treatment in preventing the tumor-like

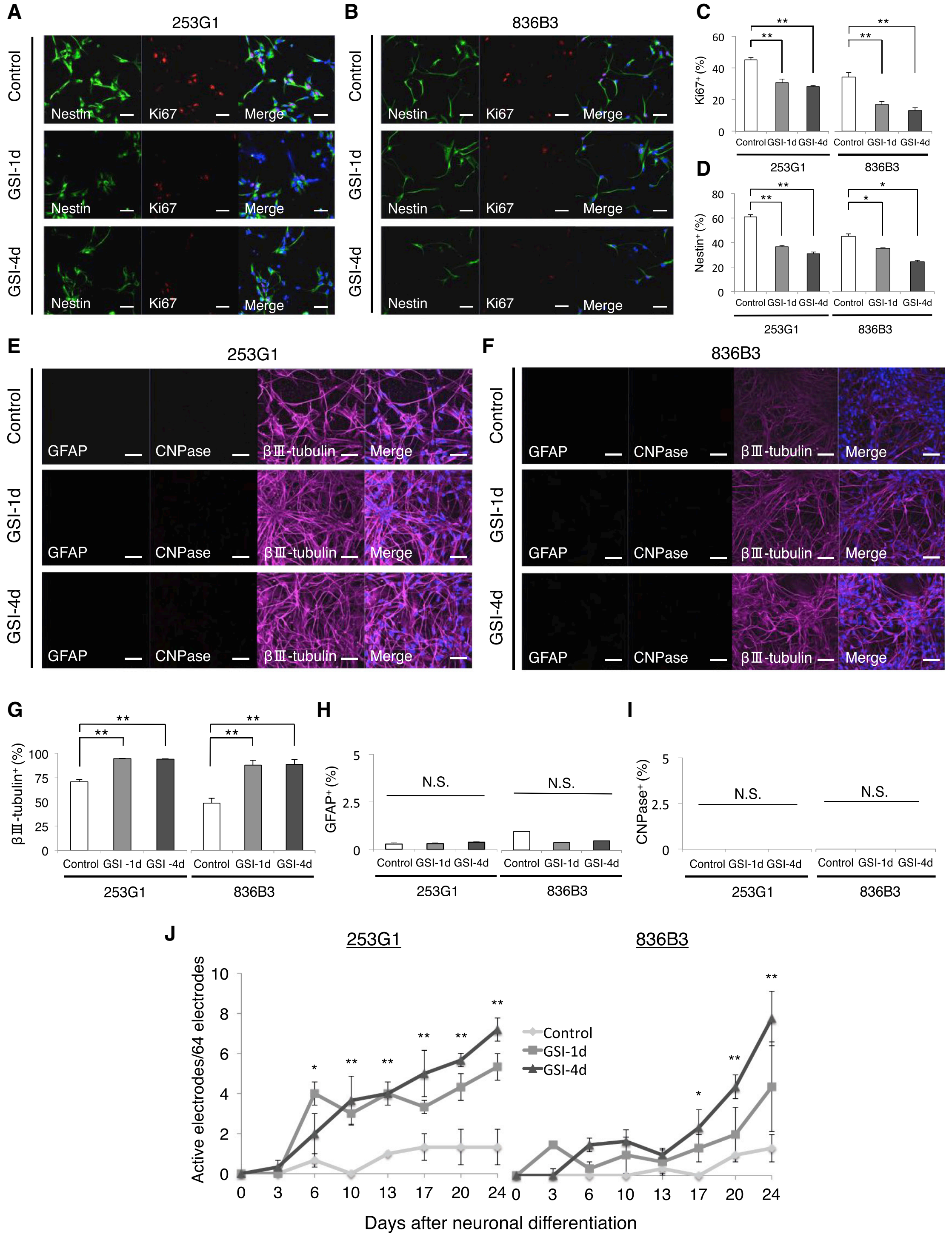

Figure 2. Neuronal Differentiation and Neuronal Maturation of hiPSC-NS/PCs Treated with or without GSI

(A and B) hiPSC-NS/PCs (with or without GSI treatment) dissociated into single cells, seeded on coverglasses, and immunostained for Ki-67 (cell proliferation marker) and Nestin (a neural stem cell marker). The nuclei were stained with Hoechst 33258. The micrographs show representative staining results for each group. Scale bars, 50 μm.

(C and D) Histograms showing the percentage of Ki-67^{+} and Nestin^{+} cells (n = 5 independent experiments).

(legend continued on next page)

overgrowth of the graft-derived cells. A previous study by our group showed that 253G1 hiPSC-NS/PCs are characterized by tumor-like overgrowth following cell transplantation in the injured spinal cord of NOD/SCID mice (Nori et al., 2015). In fact, 253G1 hiPSC-NS/PCs tended to grow and spread at a faster rate than 836B3 hiPSC-NS/PC transplants (our unpublished data). Here, we found that inhibiting Notch signaling with the GSI promoted the neuronal differentiation and maturation of hiPSC-NS/PCs in vitro, irrespective of the culture period and the cell line (Figure 2). Thus, we pretreated the 253G1 hiPSC-NS/PCs with the GSI for 1 day before cell transplantation. To monitor the survival and growth of the transplanted cells in the mouse injured spinal cord, we lentivirally transduced the hiPSC-NS/PCs with ffLuc, a fusion protein between a fluorescent protein Venus and a firefly luciferase (Hara-Miyauchi et al., 2012), which allowed us to identify the transplanted cells by their bioluminescent luciferase signals and fluorescent Venus signals. The photon counts and the number of the hiPSC-NS/PCs were significantly correlated (r^2 = 0.998, Figures S3A–S3C). The photon counts of the transplanted hiPSC-NS/PCs decreased within the first 7 days, although they gradually increased at 14 days after cell transplantation. In the control group, the photon counts were prominently increased compared with that of the GSI$^+$ group between 28 and 89 days after cell transplantation (Figures 4A and 4B). The photon counts of the control group increased more than 10-fold from their initial values at 89 days after cell transplantation. In the GSI$^+$ group, the photon counts increased more slowly and reached a plateau at 28 days after cell transplantation. Representative results of H&E staining of spinal cords from the control group and the GSI$^+$ group after injury and transplantation are shown in Figure 4C. In the control group, the HNA$^+$ transplanted cells occupied the entire spinal cord, whereas the cells in the GSI$^+$ group did not. The volumes and sizes of the grafts that were pretreated with GSI were significantly smaller than the grafts in the control group (volume of grafts: control group 7.3 ± 1.5 mm^2, GSI$^+$ group 1.3 ± 0.6 mm^2; Figures 4D and 4E). After transplantation into the injured spinal cord, the transplanted hiPSC-NS/PCs differentiated into three neural lineages in each group, such as pan-ELAVL (Hu)$^+$ neurons, GFAP$^+$ astrocytes, and APC$^+$ oligodendrocytes. We also detected the Ki-67$^+$ and Nestin$^+$ cells (Figure 4F). Immunostaining for various cell markers was examined 89 days after transplantation and subjected to quantitative analyses to evaluate the differentiation phenotype of the transplanted cells in vivo. The proportion of pan-ELAVL (Hu)$^+$ cells was significantly increased (control group 15.6% ± 1.2%, GSI$^+$ group 51.0% ± 1.8%) in the GSI$^+$ group, although the proportion of Ki-67$^+$ cells (control group 17.9$^+$ ± 0.9%, GSI$^+$ group 3.9% ± 0.9%) and that of Nestin$^+$ cells (control group 30.3% ± 1.6%, GSI$^+$ group 5.3% ± 0.8%) were also significantly decreased compared with the control group. The proportion of GFAP$^+$ cells (control group 15.6% ± 1.1%, GSI$^+$ group 11.5% ± 3.9%) and that of APC$^+$ cells (control group 5.2$^+$ ± 1.1%, GSI$^+$ group 4.2% ± 1.0%) were not significantly different between the control group and the GSI$^+$ group (Figure 4G).

We examined triple immunostaining with HNA, βIII-tubulin, and mouse-specific Bassoon (Bsn), a presynaptic marker, to evaluate the ability of the GSI-pretreated transplanted cell-derived neurons to integrate with host neural circuitry. βIII-tubulin$^+$/HNA$^+$ cells that were transplanted in parenchymal locations were contacted by Bsn$^+$ synaptic boutons of the host neurons (Figure S4A). Moreover, triple immunostaining for HNA, βIII-tubulin, and human-specific synaptophysin (hSyn) revealed that hSyn$^+$ boutons apposed the host mouse neurons (βIII-tubulin$^+$/HNA$^-$) (Figure S4B). Furthermore, immunostaining for Notch1, activated Notch1, and Snail were examined 89 days after transplantation and subjected to quantitative analyses to confirm the level of Notch activation and EMT in the transplanted cells in vivo. In the GSI$^+$ group, the proportion of Notch1$^+$ cells (control group 43.1% ± 7.2%, GSI$^+$ group 2.5% ± 2.3%), activated Notch1$^+$ cells (control group 34.1% ± 3.2%, GSI$^+$ group 0.55% ± 0.35%), and Snail$^+$ cells (control group 36.7% ± 5.1%, GSI$^+$ group 0%) were all significantly decreased compared with that of the control group (Figures 5A–5F).

Transplantation of Tumorigenesis-/Overgrowth-Prone hiPSC-NS/PCs Pretreated with GSI Maintains Motor Functional Recovery after SCI

We evaluated locomotor function using the Basso Mouse Scale (BMS) score, rotarod testing, and treadmill gait analyses with the DigiGait system. In the control group, the

(E and F) Fourteen days after neuronal induction, the hiPSC-NS/PCs were immunostained for βIII-tubulin (a neuron marker), GFAP (an astrocyte marker), and CNPase (an oligodendrocyte marker). The nuclei were stained with Hoechst 33258. The micrographs show representative staining results for each group. Scale bars, 50 μm.
(G–I) Histograms showing the percentage of βIII-tubulin$^+$, GFAP$^+$, and CNPase$^+$ cells (n = 5 independent experiments).
(J) Daily analyses of neuronal maturation using MEA, which measures the active electrodes in each group (n = 5 independent experiments).
*p < 0.05, **p < 0.01, and not significant (N.S.) according to one-way ANOVA with the Tukey-Kramer test. Data are presented as means ± SEM.

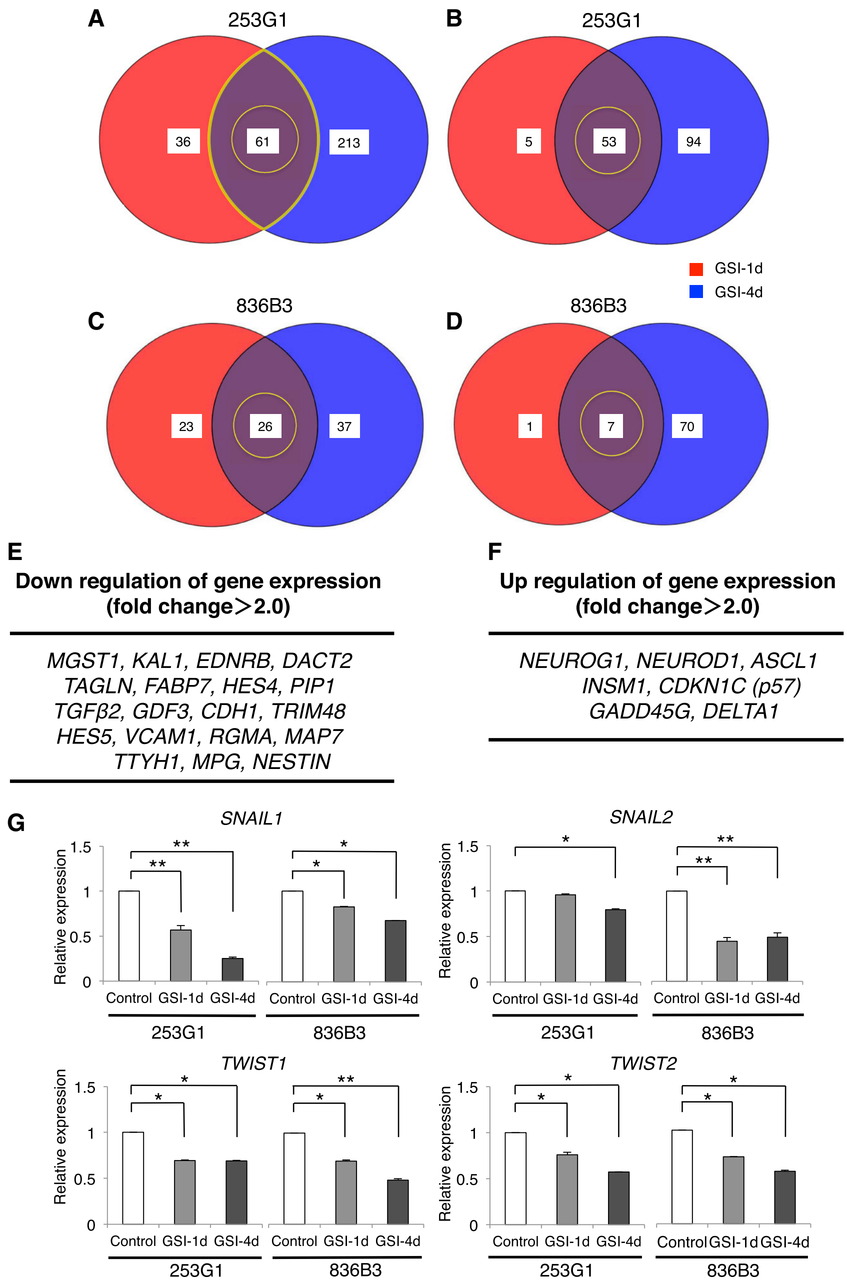

Figure 3. DNA Microarray and RT-PCR Analyses of GSI-Treated hiPSC-NS/PCs

(A) Venn diagrams showing the human genes with decreased expression in each group of GSI-treated 253G1 hiPSC-NS/PCs. Color key: red, 36 genes with decreased expression in the GSI-1d group; blue, 213 genes with decreased expression in the GSI-4d group; purple, 61 genes with decreased expression in both groups (n = 3 independent experiments).

(B) Venn diagrams showing the human genes with increased expression in each group of GSI-treated 253G1 hiPSC-NS/PCs. Color key: red, 5 genes with increased expression in the GSI-1d group; blue, 94 genes with increased expression in the GSI-4d group; purple, 53 genes with increased expression in both groups (n = 3 independent experiments).

(C) Venn diagrams showing the human genes with decreased expression in the GSI-1d and GSI-4d group of 836B3 hiPSC-NS/PCs. Color key: red, 23 genes with decreased expression in the GSI-1d group; blue, 37 genes with decreased expression in the GSI-4d group; purple, 26 genes with decreased expression in both groups (n = 3 independent experiments).

(D) Venn diagrams showing the human genes with increased expression in the GSI-1d and GSI-4d groups of 836B3 hiPSC-NS/PCs. Color key: red, 1 gene with increased expression in the GSI-1d group; blue, 70 genes with increased expression in the GSI-4d group; purple, 7 genes with increased expression in both groups (n = 3 independent experiments).

(E and F) (E) Lists showing the downregulated and (F) upregulated genes compared with control and GSI-1d and GSI-4d groups for each cell line using DNA microarrays (fold change >2.0).

(G) qRT-PCR analyses of known EMT-related genes in the GSI-1d and GSI-4d groups compared with the control group (equal to 1). The data were normalized to the reference *GAPDH* levels (n = 3 independent experiments). *$p < 0.05$ and **$p < 0.01$ according to one-way ANOVA with the Tukey-Kramer test. Data are presented as means ± SEM.

See also Figure S1.

recovery of motor function persisted for up to 35 days after transplantation, although thereafter hindlimb motor function exhibited gradual deterioration according to the BMS score. In the GSI^{+} group, significantly greater functional recovery was observed relative to the PBS group and the control group at 42 days after transplantation and was maintained thereafter (Figure 6A). The gait performance of the mice in each group was analyzed using the rotarod test and the DigiGait system at 89 days after transplantation. In the GSI^{+} group, the mice remained on the rotating rod for a significantly longer time than the mice in the PBS group and the control group (Figure 6B), and all of the mice could sufficiently walk on a treadmill at 7 cm/s to perform the test. The gait analyses also revealed a significantly longer stride length in the GSI^{+} group than in the PBS and control groups (Figure 6C).

Transplantation of Normal/Safe hiPSC-NS/PCs Pretreated with GSI Survive and Enhance Motor Functional Recovery Following SCI

Finally, we elucidated the long-term effects of GSI pretreatment on normal/safe hiPSC-NS/PCs that were transplanted into SCI model mice. Our group previously showed that 201B7 hiPSC-NS/PCs are "normal/safe" cell lines that did not display tumor-like overgrowth following transplantation in the injured spinal cord of NOD/SCID mice (Nori et al., 2011, 2015). We transplanted 201B7 hiPSC-NS/PCs with and without GSI treatment and examined their behavior after transplantation. The photon counts reached a plateau at 14 days after cell transplantation in each group (Figures 7A and 7B). The volume and size of the grafts were not significantly different between the control group and the GSI$^+$ group (graft volume: control group 2.2 ± 0.5 mm^2, GSI$^+$ group 2.1 ± 0.5 mm^2; Figures 7C–7E). In each group, the transplanted hiPSC-NS/PCs survived and did not cause tumor-like overgrowth. We examined the effects of transplantation for hiPSC-NS/PCs with and without GSI treatment on axonal regrowth after SCI by immunostaining analyses. More neurofilament 200-kDa-positive neuronal fibers and 5-hydroxytryptamine-positive serotonergic fibers were observed in the GSI$^+$ group than in the PBS and control groups (Figures S5A and S5B). In the GSI$^+$ group, there were also significantly more growth-associated protein 43-positive fibers in the ventral region 1 mm caudal to the lesion epicenter than in the PBS and control groups (Figures S5C and S5D).

The recovery of motor function persisted for up to 89 days after transplantation in each group compared with the PBS group. Furthermore, in the GSI$^+$ group, significantly greater functional recovery occurred compared with the control group at 35 days after transplantation (Figure 7F). In the GSI$^+$ group, the mice remained on the rotating rod for a significantly longer time those in the PBS and control groups (Figure 7G). In the treadmill gait analyses, significant differences were not observed in stride length between the control group and the GSI$^+$ group (Figure 7H).

DISCUSSION

In the present study, we report seven major findings. First, after the hiPSC-NS/PCs were treated with the GSI, the number of cells, the size of neurospheres, the proportion of cells in the S phase of the cell cycle, and the number of Ki-67$^+$ and Nestin$^+$ cells determined by immunostaining were all significantly decreased compared with that of the control group. Second, the proportion of βIII-tubulin$^+$ neuronal cells was significantly increased in the GSI-treated group compared with the control (Figure 1), and an MEA assay showed that the average number of active electrodes occurred significantly earlier and exhibited much more neuronal activity than the control group (Figure 2J). Third, the gene expression profiles of the GSI-treated hiPSC-NS/PCs obtained from the DNA microarray and RT-PCR showed that the expression of cell-cycle-related genes, mature neuronal marker-related genes, and p53-dependent genes was increased compared with that of the control group, which is consistent with decreases in pluripotency and self-renewal marker genes (Figure 3). Fourth, after transplantation of the hiPSC-NS/PCs in animal models of SCI, the photon counts of the transplanted cells increased more slowly and reached a plateau at 28 days in the GSI$^+$ groups as determined by bioluminescence (BLI) (Figures 4A and 4B). Fifth, in the GSI$^+$ group, the proportion of pan-ELAVL (Hu)$^+$ cells was significantly increased, although the proportion of Ki-67$^+$ cells, Nestin$^+$ cells, Notch1$^+$ cells, activated Notch1$^+$ cells, and Snail$^+$ cells was significantly decreased compared with that of the control group (Figures 4 and 5). Sixth, in the long-term observations, the control group showed deteriorated motor function accompanied by tumor-like overgrowth. However, the GSI$^+$ group maintained functional recovery and reduced the overgrowth of transplanted cells by inhibiting cell proliferation (Figure 6). Seventh, after transplantation of a normal/safe cell line, the grafts that were pretreated with GSI also survived and did not show tumor-like overgrowth, which caused motor functional recovery after SCI (Figure 7). Taken together, these results suggest that the GSI treatment induces the differentiation of hiPSC-NS/PCs into a more mature state with limited proliferation and contributes to functional recovery without tumor-like overgrowth in the injured spinal cord. We believe that this strategy improves the safety of hiPSC-NS/PC transplantation therapy for SCI.

Previous studies have reported that Notch signaling controls the induction of neural stem cells in vitro and in vivo during differentiation (Crawford and Roelink, 2007; Nelson et al., 2007). As established by Ogura et al. (2013), inhibition of this signaling pathway promotes the neuronal differentiation of neural progenitor cells derived from human iPSCs. Our in vitro experiments confirmed that by inhibiting Notch signaling with the GSI, a greater number of NS/PCs derived from two lines of human iPSCs with tumorigenesis-/overgrowth-prone characters (253G1 and 836B3) are induced to undergo neuronal differentiation and maturation, although they present limited proliferation.

Nelson et al. (2007) showed treatments with a GSI (DAPT) lasting at least 6 hr commits NS/PCs to neuronal differentiation, although *HES5* expression is reduced after 3 hr. In the present study, significant differences were not

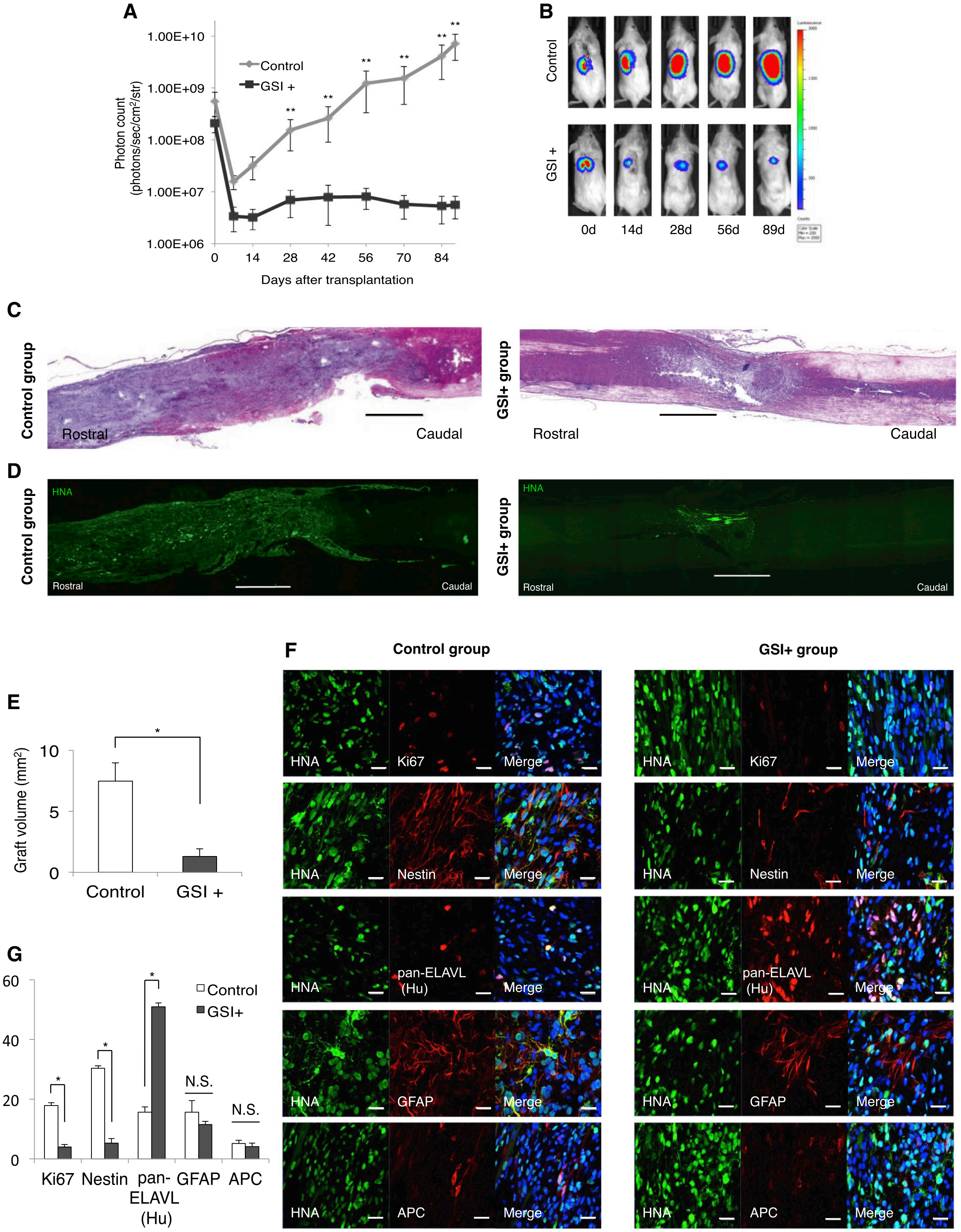

Figure 4. Long-Term Observation after Transplantation of GSI-Pretreated 253G1 hiPSC-NS/PCs

(A) Quantitative analyses of the photon counts derived from the transplanted cells until 89 days post transplantation (control group, n = 10 mice; GSI^{+} group, n = 10 mice).

(B) Representative images of mice at 0, 14, 28, 56, and 89 days after transplantation of 253G1 hiPSC-NS/PCs that were pretreated with or without GSI.

(C) Representative H&E-stained images of the spinal cord from each group at 89 days after transplantation. Scale bars, 1,000 μm.

(legend continued on next page)

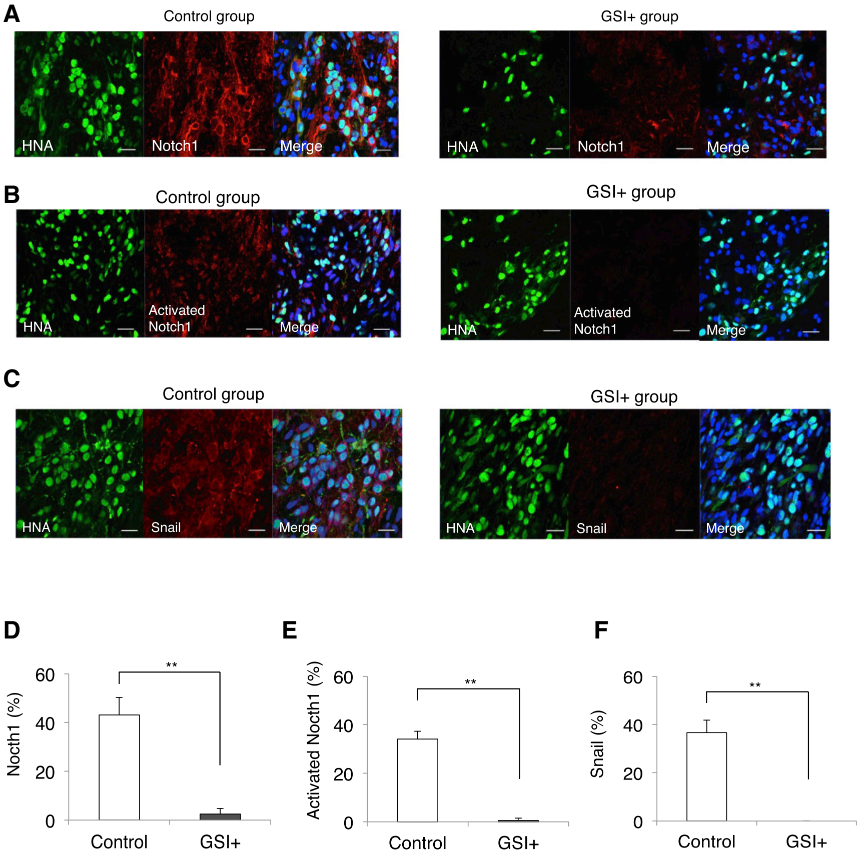

Figure 5. Level of Notch and EMT Activation in the Transplanted Cells Following Long-Term Observations In Vivo

(A–C) Representative images of immunohistochemical staining show the results for each group. The HNA^+ transplanted cells were stained with Notch1, activated Notch1, and Snail. Scale bars, 20 μm.

(D–F) Histograms showing the percentages of cell-type-specific, marker-positive cells among the HNA^+ transplanted cells at 89 days after transplantation (control group, n = 10 mice; GSI^+ group, n = 10 mice). $^{**}p < 0.01$ according to the Wilcoxon rank-sum test. Data are presented as means ± SEM.

observed between the GSI-1d and GSI-4d group in hiPSC-NS/PC proliferation and differentiation, suggesting that inhibition of Notch signaling with a GSI promotes the neuronal differentiation and maturation of hiPSC-NS/PCs regardless of the culture period or cell lines.

Nori et al. (2011) showed that the 201B7 hiPSC-NS/PC-transplanted group exhibited significantly better functional recovery than the PBS-injected group more than 21 days after SCI as measured by the BMS score. In this study, the GSI^+ group of 253G1 hiPSC-NS/PC transplants ("bad" clone) exhibited confirmed functional recovery more than 21 days after SCI (BMS score: PBS group 2.5 ± 0.3 at 12 days post transplantation and 3.0 ± 0.1 at 89 days post transplantation; GSI^+ group 3.8 ± 0.7 at 12 days post transplantation and 4.8 ± 0.4 at 89 days post transplantation). Borghese et al. (2010) showed that the neural stem cell derived from human embryonic cells pretreated with DAPT were strongly positive for human synaptophysin, and exhibited strong human doublecortin staining compared with untreated cells after transplantation. In this study, the GSI^+ group of 201B7 hiPSC-NS/PC transplants ("good" clone) exhibited a significantly greater tendency to enhance the axonal regrowth in the mouse injured spinal cord, which are likely to have resulted in improved motor function compared with the control group at 26 days after transplantation. The improved

(D) Representative immunohistological images of HNA^+ cells showing a graft from each group at 89 days after transplantation. Scale bars, 1,000 μm.

(E) Graft volumes of hiPSC-NS/PCs were measured and estimated for each group of SCI at 89 days after transplantation (control group, n = 10 mice; GSI^+ group, n = 10 mice).

(F) Representative images of the immunohistochemical staining show the results for each group. The HNA^+ transplanted cells were stained with Ki-67, Nestin, pan-ELAVL (Hu), GFAP, and APC. Scale bar, 20 μm.

(G) Percentages of cell-type-specific, marker-positive cells among the HNA^+ transplanted cells at 89 days after transplantation (control group, n = 10 mice; GSI^+ group, n = 10 mice).

$^*p < 0.05$, $^{**}p < 0.01$, and not significant (N.S.) according to one-way ANOVA with the Tukey-Kramer test (G) or Wilcoxon rank-sum test (A and E). Data are presented as means ± SEM.

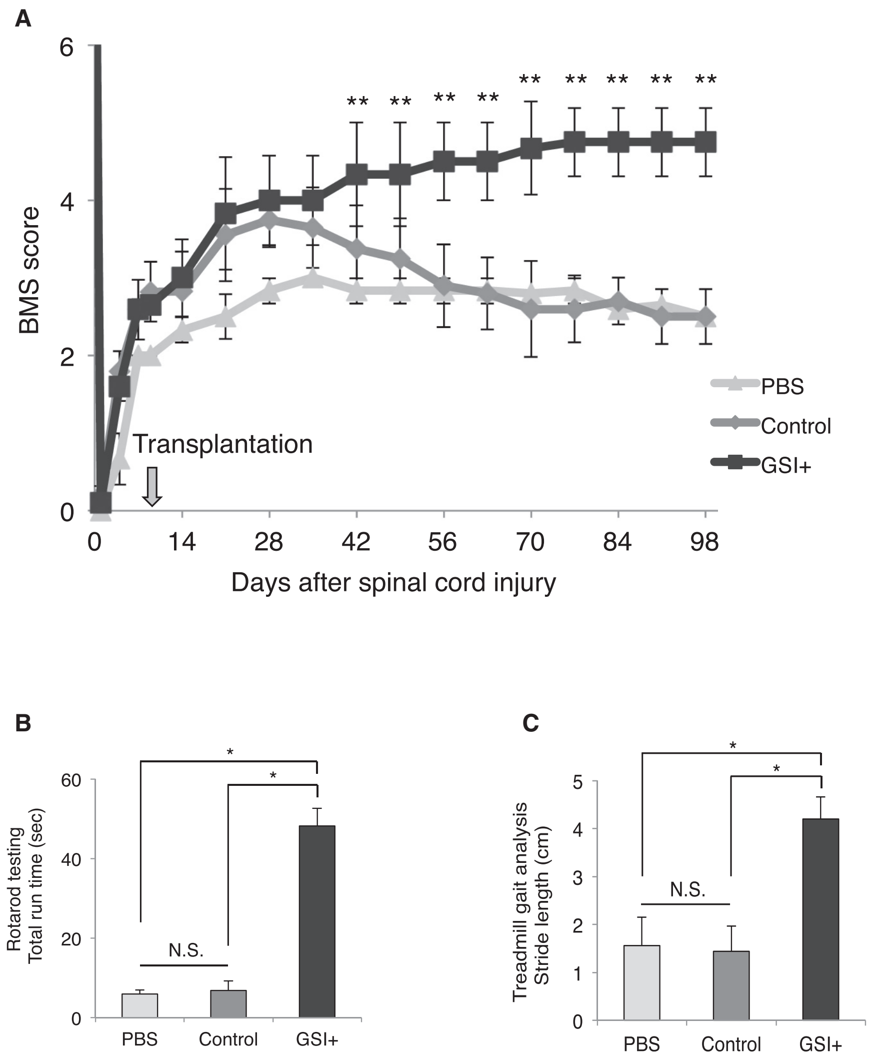

Figure 6. Long-Term Motor Function Analyses after Transplantation of GSI-Pretreated hiPSC-NS/PCs

(A) Comparison of the BMS scores among the PBS, control, and GSI$^+$ groups. Motor function in the hind limbs was assessed weekly for up to 89 days after transplantation using the BMS score (PBS group, n = 10; control group, n = 10; GSI$^+$ group, n = 10 mice).

(B) Comparison of the rotarod test among the PBS, control, and GSI$^+$ groups. The rotarod test was performed at 89 days after transplantation. Histograms show the total run time (PBS group, n = 10; control group, n = 10; GSI$^+$ group, n = 10 mice).

(C) Comparison of the stride lengths among the PBS, control, and GSI$^+$ groups. Treadmill gait analyses were performed at 89 days after transplantation using the DigiGait system. Histograms show the stride length (PBS group, n = 10; control group, n = 10; GSI$^+$ group, n = 10 mice).

*$p < 0.05$, **$p < 0.01$, and not significant (N.S.) according to two-way repeated-measures ANOVA with the Tukey-Kramer test (A) or one-way ANOVA with the Tukey-Kramer test (B and C). Data are presented as means ± SEM.

motor function was maintained after this point in the GSI$^+$ group (BMS score: control group 3.8 ± 0.2 at 26 days post transplantation and 4.4 ± 0.5 at 89 days post transplantation; GSI$^+$ group 4.3 ± 0.5 at 26 days post transplantation and 4.9 ± 0.3 at 89 days post transplantation). It is possible that pretreatment with GSI might have other mechanisms besides axonal regrowth, and could have a clinically meaningful effect and improve human iPSC-based transplantation for SCI.

Improved cell quality and safety, particularly with respect to the risk of tumor-like overgrowth, will be crucially important for any clinical use of hiPSC-NS/PCs. Okada et al. (2005) revealed that the photon counts measured by BLI analyses were significantly proportional to the number of the transplanted cells in vivo. Nori et al. (2015) indicated that some mice transplanted with 253G1 hiPSC-NS/PCs showed temporary motor function recovery for up to 47 days after transplantation; however, the photon counts of the transplanted cells increased more than 10-fold from its initial value, and these mice also developed tumor-like overgrowth and deterioration of motor function at 103 days after transplantation. Therefore, the photon counts measured by BLI analyses could be useful for the diagnosis for detecting tumor-like overgrowth in the mouse injured spinal cords. In the present study, the control group (i.e., the group without GSI pretreatment) showed a rapid increase in the photon count of the transplanted cells and improvements in hindlimb motor function, which subsequently deteriorated gradually upon long-term observations. However, in the GSI$^+$ groups, the photon counts increased more slowly and reached a plateau after transplantation. Greater functional recovery was also observed, and was maintained at significantly higher levels compared with the control group. In addition, we previously reported that the size of the tumors formed by the transplanted hiPSC-NS/PCs in the injured

spinal cord correlated with the proportion of Nestin$^+$ cells in the graft (Nori et al., 2015). The proportion of transplanted Nestin$^+$ cells decreased from 10.7% ± 2.2% at 47 days to 7.5% ± 1.0% at 103 days after transplanting 201B7 hiPSC-NS/PCs, resulting in no tumor-like overgrowth. The proportion of Nestin$^+$ cells increased from 19.6% ± 0.5% at 47 days to 33.1% ± 7.4% at 103 days after transplanting 253G1 hiPSC-NS/PCs. Therefore, we suggest that differentiation-resistant Nestin$^+$ cells proliferated over time and formed tumors. However, the present study showed that the proportion of transplanted Nestin$^+$ cells in the control group increased to 30.3% ± 1.6% at 89 days after transplantation and resulted in tumor-like overgrowth, whereas the proportion of Nestin$^+$ cells in the GSI$^+$ group decreased to 5.3% ± 0.8% at 89 days after transplantation and did not show evidence of tumor-like overgrowth. The proportion of Ki-67$^+$ cells in the control group was significantly increased to 17.9% ± 0.9% at 89 days after transplantation compared with the GSI$^+$ group. Furthermore, the proportion of pan-ELAVL (Hu)$^+$ neuronal cells in the GSI$^+$ groups significantly increased to 51.0% ± 1.8% compared with the control group at 89 days after transplantation, although significant differences were not observed in glial differentiation. Most of the GSI-pretreated, transplanted βIII-tubulin$^+$/HNA$^+$ cell-derived neurons were co-localized with Bsn$^+$ synaptic boutons of the host neurons, and hSyn$^+$ boutons were apposed to βIII-tubulin$^+$/HNA$^-$ host mouse neurons. These findings indicate that the proportion of proliferating/undifferentiating cells increases over time and induces tumor-like overgrowth in the vertebral canal of the mice. Therefore, the motor functional recovery observed in the control group subsequently deteriorated gradually upon long-term observations. However, pretreatment with GSI differentiated nearly all of these cells into mature neurons, which were further integrated into the reconstructed host neuronal network, where they formed synapses and prevented tumor-like overgrowth, even in mice transplanted with the "bad" clone, after observations for long periods. Therefore, significantly greater motor functional recovery was maintained compared with the control group.

The activation of Notch1 has been found to contribute to the invasion and metastasis of cancer via the EMT. In addition, Saad et al. (2010) showed that the overexpression of *NOTCH1* induces the expression of *SNAIL*. In the present study, after GSI treatment the expression of *NOTCH1*, *SNAIL1*, and *SNAIL2* was downregulated in vitro compared with the control group. Furthermore, the proportion of Notch1$^+$, activated Notch1$^+$, and Snail$^+$ cells was also significantly decreased after transplantation compared with the control group, which indicated that the presence of activated Notch1 on the transplanted cells reflected the γ-secretase-dependent activation of Notch1 in vivo. Therefore, the GSI pretreatment could reduce the acquisition of EMT and inhibit the activation of Notch1 and Snail, which induced the transplanted cells to undergo neuronal maturation without tumor-like overgrowth upon transplantation of hiPSC-NS/PCs for SCI. However, Kopan and Ilagan (2004) showed that other signaling molecules act as targets for GSI. Thus, we cannot rule out the possibility that the GSI-induced inhibition of other signaling pathways, such as the expression of EMT-related genes, contributed to these results.

Longer differentiation periods have been shown to decrease the incidence of tumors. Alternatively, the selection of more mature cells using fluorescence-activated or magnetic-activated cell sorting, introduction of suicide genes, and irradiation of cells before transplantation may also help inhibit tumor-like overgrowth. However, these methods require long periods and more expensive processes to prepare the donor cells. In contrast, GSI pretreatment is a simple and inexpensive method that could be useful for improving the safety of hiPSC-NS/PCs transplantation therapy in SCI.

In conclusion, we confirmed that the GSI-treated hiPSC-NS/PCs exhibited a reduced proportion of dividing cells and increased neuronal maturation in vitro, and prevented the tumor-like overgrowth of transplanted cells by inhibiting cell proliferation, thereby resulting in safe and long-lasting functional recovery in vivo. Pretreatment of hiPSC-NS/PCs with GSI can improve the safety of hiPSC-NS/PC transplantation therapy for SCI.

EXPERIMENTAL PROCEDURES

Cell Culture, hiPSC-NS/PC-Derived NS/PC Formation Assay, Neuronal Differentiation Analyses, and Lentiviral Transduction

The cell culture, hiPSC-NS/PC-derived NC/PC formation assay, neuronal differentiation analyses of several human iPSCs, and lentiviral transduction of neurospheres were performed as previously described (Nori et al., 2011, 2015; Okada et al., 2008). In brief, three hiPSC-NS/PCs lines were dissociated and infected with a lentivirus expressing ffLuc, a firefly luciferase fusion protein under the control of the EF promoter, to enable the detection of transplanted cells through their strong bioluminescent ffLuc signals in live SCI mice and in fixed spinal cord sections. Detailed methods are described in Supplemental Experimental Procedures.

Treatment of hiPSC-NS/PCs with GSI

One of the small-molecule GSIs, N-[N-(3,5-difluorophenacetyl)-l-alanyl]-S-phenylglycine t-butyl ester (DAPT; Sigma-Aldrich), is a potent nontransition-state analog inhibitor of γ-secretase that is thought to interact with the same active site between presenilin-1 heterodimers in γ-secretase complexes. DAPT was dissolved in DMSO at a final concentration of 10 μM. The highest

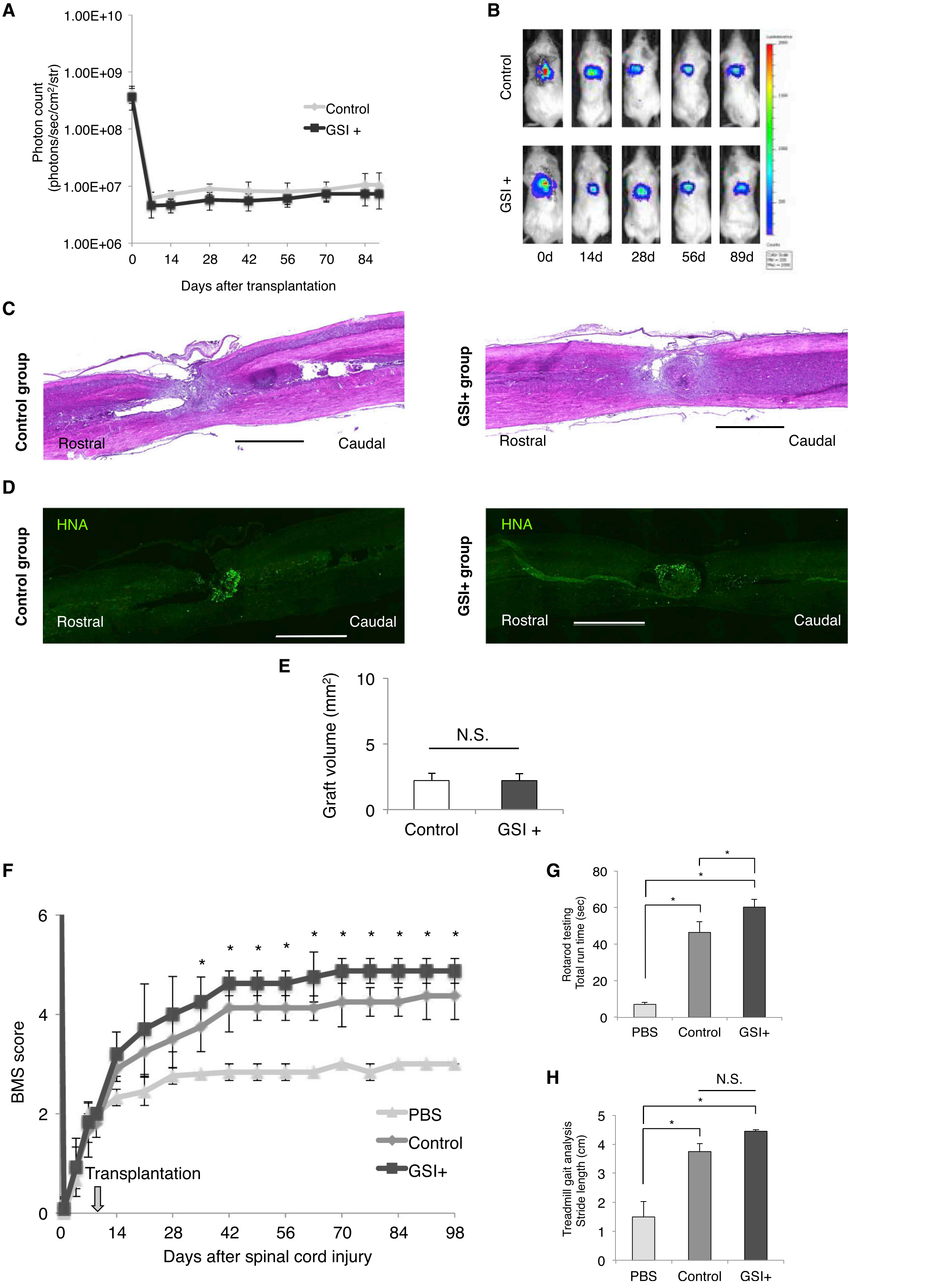

(legend on next page)

concentration of this molecule that effectively inhibited the division of hiPSC-NS/PCs without precipitating in culture medium or producing toxic effects was determined in preliminary experiments. The hiPSC-NS/PCs were cultured in vitro with GSI for 1 day in the GSI-1d group, 4 days in the GSI-4d group, and without GSI in the control group. For the neuronal differentiation, proliferation, and cell transplantation assays, the hiPSC-NS/PCs were used after the fifth passage.

Flow Cytometric Analyses

In the cell-cycle analyses, the hiPSC-NS/PCs were dissociated into single cells and double immunofluorescence staining was performed with the Click-iT Plus EdU Flow Cytometry Assay Kit (Life Technologies). In the apoptosis analyses, double immunofluorescence staining was performed using the primary antibodies anti-allophycocyanin (APC)-labeled annexin V (561012) and 7-amino-actinomycin D (7-AAD, 559925) (BD Biosciences). The cells were stained with a mixture of these primary antibodies for 15 min at 37°C. Detailed methods are described in Supplemental Experimental Procedures.

Microelectrode Array

The MEA was performed as previously described (Köhling et al., 2005), and detailed methods are provided in Supplemental Experimental Procedures.

DNA Microarray Analyses

DNA microarray analyses were performed as previously described (Parinya et al., 2014), and detailed methods are provided in Supplemental Experimental Procedures. GO accessions are listed and shown in Tables S1 and S2.

qRT-PCR

RNA isolation and RT-PCR were performed as previously described (Nori et al., 2011, 2015; Okada et al., 2008), and detailed protocols are provided in Supplemental Experimental Procedures.

SCI Animal Model and hiPSC-NS/PC Transplantation

Contusive SCI was induced at the level of the tenth thoracic spinal vertebra in the spinal cords of adult female NOD-SCID mice. Nine days after SCI, hiPSC-NS/PCs that had been pretreated with or without GSI (5×10^5 cells/2 μL) were transplanted into the lesion epicenter of each mouse. All experiments were performed in accordance with the Guidelines for the Care and Use of Laboratory Animals of Keio University (Assurance No. 13020) and the NIH Guide for the Care and Use of Laboratory Animals. Detailed methods are provided in Supplemental Experimental Procedures.

Histological Analysis

Histological analyses were performed at 89 days after transplantation. Spinal cord sections were histologically evaluated by H&E staining and immunohistochemistry (IHC). Detailed protocols are provided in Supplemental Experimental Procedures.

Behavioral Analyses

The motor function of each mouse in the PBS group, the control group, and the GSI$^+$ group was evaluated weekly for up to 98 days after injury using the BMS scores (Basso et al., 2006). Detailed methods are provided in Supplemental Experimental Procedures.

Statistical Analysis

All data are reported as mean ± SEM. A Wilcoxon rank-sum test was used to evaluate the differences between groups with respect to the BLI analyses, H&E staining, and IHC results. One-way ANOVA followed by the Tukey-Kramer test for multiple comparisons was used to evaluate the differences in the following: number of living cells; sphere size; flow cytometric analyses of the cell cycle/apoptosis assays; neuronal differentiation analyses; MEA; DNA microarray analyses; RT-PCR gene expression profile analyses; and the rotarod and DigiGait results. Two-way repeated-measures ANOVA tests followed by the Tukey-Kramer test were used for the BMS analyses. p Values of <0.05 or <0.01 were considered to indicate statistical significance.

Figure 7. Long-Term Observations after Transplantation of GSI-Pretreated 201B7 hiPSC-NS/PCs

(A) Quantitative analyses of the photon counts derived from the transplanted cells until 89 days post transplantation (control group, n = 10 mice; GSI$^+$ group, n = 10 mice).

(B) Representative images of mice at 0, 14, 28, 56, and 89 days after transplantation of 201B7 hiPSC-NS/PCs that were pretreated with or without GSI.

(C) Representative H&E-stained images of the spinal cords from each group at 89 days after transplantation. Scale bars, 1,000 μm.

(D) Representative immunohistological images of HNA$^+$ cells, showing a graft from each group at 89 days after transplantation. Scale bars, 1,000 μm.

(E) Graft volumes of the hiPSC-NS/PCs were measured and estimated for each group of SCI model animals at 89 days after transplantation (control group, n = 10 mice; GSI$^+$ group, n = 10 mice).

(F) Comparison of the BMS scores among the PBS, control, and GSI$^+$ groups. Motor function in the hind limbs was assessed weekly for up to 89 days after transplantation using the BMS score (PBS group, n = 10; control group, n = 10; GSI$^+$ group, n = 10 mice).

(G) Comparison of the rotarod test among the PBS, control, and GSI$^+$ groups. The rotarod test was performed at 89 days after transplantation. Histograms show the total run time (PBS group, n = 10; control group, n = 10; GSI$^+$ group, n = 10 mice).

(H) Comparison of the stride lengths among the PBS, control, and GSI$^+$ groups. Treadmill gait analyses were performed at 89 days after transplantation using the DigiGait system. Histograms show the stride lengths (PBS group, n = 10; control group, n = 10; GSI$^+$ group, n = 10 mice).

*$p < 0.05$ and not significant (N.S.) according to the Wilcoxon rank-sum test (A and E), two-way repeated-measures ANOVA with the Tukey-Kramer test (F), or one-way ANOVA with the Tukey-Kramer test (G and H). Data are presented as means ± SEM.

ACCESSION NUMBERS

The DNA microarray data have been submitted to the NCBI Gene Expression Omnibus under accession number GEO: GSE76556.

SUPPLEMENTAL INFORMATION

Supplemental Information includes Supplemental Experimental Procedures, five figures, and two tables and can be found with this article online at http://dx.doi.org/10.1016/j.stemcr.2016.08.015.

AUTHOR CONTRIBUTIONS

T.O. designed the project, performed most of the experiments, interpreted the data, and wrote the manuscript with technical assistance from A.I. and J.K. G.I., S.K., Y.N., K.S., M.O., T.I., K.M., and M.M. provided experimental support and ideas for the project. M.N. and H.O. designed the studies, supervised the overall project, and prepared the final manuscript.

ACKNOWLEDGMENTS

We appreciate the assistance of Drs. N. Nagoshi, K. Kojima, and S. Ito, who are all members of the spinal cord research team in the Department of Orthopedic Surgery, Keio University School of Medicine, Tokyo, Japan. We also thank Ms. T. Harada, Ms. R. Kashiwagi, and Ms. K. Yasutake for their assistance with the experiments and animal care. We thank Prof. Douglass Sipp (Keio University) for providing invaluable comments on the manuscript. This work was supported by Research Center Network for Realization of Regenerative Medicine from the Japan Science and Technology Agency (JST), the Japan Agency for Medical Research and Development (AMED) (grant no. 15bm0204001h0003 to H.O. and M.N.), and in part by a medical research grant on traffic accidents from The General Insurance Association of Japan (grant no. 15-1-42). H.O. is a compensated scientific consultant of San Bio, Co., Ltd.

Received: January 11, 2016
Revised: August 23, 2016
Accepted: August 23, 2016
Published: September 22, 2016

REFERENCES

Basso, D.M., Fisher, L.C., Anderson, A.J., Jakeman, L.B., McTigue, D.M., and Popovich, P.G. (2006). Basso Mouse Scale for locomotion detects differences in recovery after spinal cord injury in five common mouse strains. J. Neurotrauma *23*, 635–659.

Borghese, L., Dolezalova, D., Opitz, T., Haupt, S., Leinhaas, A., Steinfarz, B., Koch, P., Edenhofer, F., Hampl, A., and Brüstle, O. (2010). Inhibition of Notch signaling in human embryonic stem cell-derived neural stem cells delays G1/S phase transition and accelerates neuronal differentiation in vitro and in vivo. Stem Cells *28*, 955–964.

Crawford, T.Q., and Roelink, H. (2007). The notch response inhibitor DAPT enhances neuronal differentiation in embryonic stem cell-derived embryoid bodies independently of sonic hedgehog signaling. Dev. Dyn. *236*, 886–892.

Cummings, B.J., Uchida, N., Tamaki, S.J., Salazar, D.L., Hooshmand, M., Summers, R., Gage, F.H., and Anderson, A.J. (2005). Human neural stem cells differentiate and promote locomotor recovery in spinal cord-injured mice. Proc. Natl. Acad. Sci. USA *102*, 14069–14074.

Falk, A., Koch, P., Kesavan, J., Takashima, Y., Ladewig, J., Alexander, M., Wiskow, O., Tailor, J., Trotter, M., Pollard, S., et al. (2012). Capture of neuroepithelial-like stem cells from pluripotent stem cells provides a versatile system for in vitro production of human neurons. PLoS One *7*, e29597.

Fujimoto, Y., Abematsu, M., Falk, A., Tsujimura, K., Sanosaka, T., Juliandi, B., Semi, K., Namihira, M., Komiya, S., Smith, A., et al. (2012). Treatment of a mouse model of spinal cord injury by transplantation of human induced pluripotent stem cell-derived long-term self-renewing neuroepithelial-like stem cells. Stem Cells *30*, 1163–1173.

Hara-Miyauchi, C., Tsuji, O., Hanyu, A., Okada, S., Yasuda, A., Fukano, T., Akazawa, C., Nakamura, M., Imamura, T., Matsuzaki, Y., et al. (2012). Bioluminescent system for dynamic imaging of cell and animal behavior. Biochem. Biophys. Res. Commun. *419*, 188–193.

Hofstetter, C.P., Holmström, N.A., Lilja, J.A., Schweinhardt, P., Hao, J., Spenger, C., Wiesenfeld-Hallin, Z., Kurpad, S.N., Frisén, J., and Olson, L. (2005). Allodynia limits the usefulness of intraspinal neural stem cell grafts; directed differentiation improves outcome. Nat. Neurosci. *8*, 346–353.

Iwanami, A., Kaneko, S., Nakamura, M., Kanemura, Y., Mori, H., Kobayashi, S., Yamasaki, M., Momoshima, S., Ishii, H., Ando, K., et al. (2005). Transplantation of human neural stem cells for spinal cord injury in primates. J. Neurosci. Res. *80*, 182–190.

Kobayashi, Y., Okada, Y., Itakura, G., Iwai, H., Nishimura, S., Yasuda, A., Nori, S., Hikishima, K., Konomi, T., Fujiyoshi, K., et al. (2012). Pre-evaluated safe human iPSC-derived neural stem cells promote functional recovery after spinal cord injury in common marmoset without tumorigenicity. PLoS One *7*, e52787.

Kopan, R., and Ilagan, M.X. (2004). Gamma-secretase: proteasome of the membrane? Nat. Rev. Mol. Cell Biol. *5*, 499–504.

Kumagai, G., Okada, Y., Yamane, J., Nagoshi, N., Kitamura, K., Mukaino, M., Tsuji, O., Fujiyoshi, K., Katoh, H., Okada, S., et al. (2009). Roles of ES cell-derived gliogenic neural stem/progenitor cells in functional recovery after spinal cord injury. PLoS One *4*, e7706.

Köhling, R., Melani, R., Koch, U., Speckmann, E.J., Koudelka-Hep, M., Thiébaud, P., and Balestrino, M. (2005). Detection of electrophysiological indicators of neurotoxicity in human and rat brain slices by a three-dimensional microelectrode array. Altern. Lab. Anim. *33*, 579–589.

Miura, K., Okada, Y., Aoi, T., Okada, A., Takahashi, K., Okita, K., Nakagawa, M., Koyanagi, M., Tanabe, K., Ohnuki, M., et al. (2009). Variation in the safety of induced pluripotent stem cell lines. Nat. Biotechnol. *27*, 743–745.

Nelson, B.R., Hartman, B.H., Georgi, S.A., Lan, M.S., and Reh, T.A. (2007). Transient inactivation of Notch signaling synchronizes differentiation of neural progenitor cells. Dev. Biol. *304*, 479–498.

Nori, S., Okada, Y., Yasuda, A., Tsuji, O., Takahashi, Y., Kobayashi, Y., Fujiyoshi, K., Koike, M., Uchiyama, Y., Ikeda, E., et al. (2011). Grafted human-induced pluripotent stem-cell-derived neurospheres promote motor functional recovery after spinal cord injury in mice. Proc. Natl. Acad. Sci. USA *108*, 16825–16830.

Nori, S., Okada, Y., Nishimura, S., Sasaki, T., Itakura, G., Kobayashi, Y., Francois, R.H., Shimizu, A., Koya, I., Yoshida, R., et al. (2015). Long-term safety issues of iPSC-based cell therapy in a spinal cord injury model: oncogenic transformation with epithelial-mesenchymal transition. Stem Cell Rep. *4*, 360–373.

Ogawa, Y., Sawamoto, K., Miyata, T., Miyao, S., Watanabe, M., Nakamura, M., Bregman, B.S., Koike, M., Uchiyama, Y., Toyama, Y., and Okano, H. (2002). Transplantation of in vitro-expanded fetal neural progenitor cells results in neurogenesis and functional recovery after spinal cord contusion injury in adult rats. J. Neurosci. Res. *69*, 925–933.

Ogura, A., Morizane, A., Nakajima, Y., Miyamoto, S., and Takahashi, J. (2013). γ-Secretase inhibitors prevent overgrowth of transplanted neural progenitors derived from human-induced pluripotent stem cells. Stem Cell Dev. *22*, 374–382.

Okada, Y., Shimazaki, T., Sobue, G., and Okano, H. (2004). Retinoic acid concentration dependent acquisition of neural cell identity during in vitro differentiation of mouse embryonic stem cells. Dev. Biol. *275*, 124–142.

Okada, S., Ishii, K., Yamane, J., Iwanami, A., Ikegami, T., Katoh, H., Iwamoto, Y., Nakamura, M., Miyoshi, H., Okano, H.J., et al. (2005). In vivo imaging of engrafted neural stem cells: its application in evaluating the optimal timing of transplantation for spinal cord injury. FASEB J. *19*, 1839–1841.

Okada, Y., Matsumoto, A., Shimazaki, T., Enoki, R., Koizumi, A., Ishii, S., Itoyama, Y., Sobue, G., and Okano, H. (2008). Spatiotemporal recapitulation of central nervous system development by murine embryonic stem cell-derived neural stem/progenitor cells. Stem Cells *26*, 3086–3098.

Okano, H., Nakamura, M., Yoshida, K., Okada, Y., Tsuji, O., Nori, S., Ikeda, E., Yamanaka, S., and Miura, K. (2013). Steps toward safe cell therapy using induced pluripotent stem cells. Circ. Res. *112*, 523–533.

Parinya, N., Carina, L., Kartiek, K., Riikka, L., Harri, L., Ritta, L., Karolina, L., Hataiwan, C., Timo, O., Tiom, T., et al. (2014). Notch signaling regulates the differentiation of neural crest from human pluripotent stem cells. J. Cell Sci. *127*, 2083–2094.

Saad, S., Stanners, S.R., Yong, R., Tang, O., and Pollock, C.A. (2010). Notch mediated epithelial to mesenchymal transformation is associated with increased expression of the Snail transcription factor. Int. J. Biochem. Cell Biol. *42*, 1115–1122.

Salazar, D.L., Uchida, N., Hamers, F.P., Cummings, B.J., and Anderson, A.J. (2010). Human neural stem cells differentiate and promote locomotor recovery in an early chronic spinal cord injury NOD-scid mouse model. PLoS One *5*, e12272.

Tsuji, O., Miura, K., Okada, Y., Fujiyoshi, K., Mukaino, M., Nagoshi, N., Kitamura, K., Kumagai, G., Nishino, M., Tomisato, S., et al. (2010). Therapeutic potential of appropriately evaluated safe-induced pluripotent stem cells for spinal cord injury. Proc. Natl. Acad. Sci. USA *107*, 12704–12709.

Yasuda, A., Tsuji, O., Shibata, S., Nori, S., Takano, M., Kobayashi, Y., Takahashi, Y., Fujiyoshi, K., Hara, C.M., Miyawaki, A., et al. (2011). Significance of remyelination by neural stem/progenitor cells transplanted into the injured spinal cord. Stem Cells *29*, 1983–1994.

Stem Cell Reports, Volume *7*

Supplemental Information

Pretreatment with a γ-Secretase Inhibitor Prevents Tumor-like Overgrowth in Human iPSC-Derived Transplants for Spinal Cord Injury

Toshiki Okubo, Akio Iwanami, Jun Kohyama, Go Itakura, Soya Kawabata, Yuichiro Nishiyama, Keiko Sugai, Masahiro Ozaki, Tsuyoshi Iida, Kohei Matsubayashi, Morio Matsumoto, Masaya Nakamura, and Hideyuki Okano

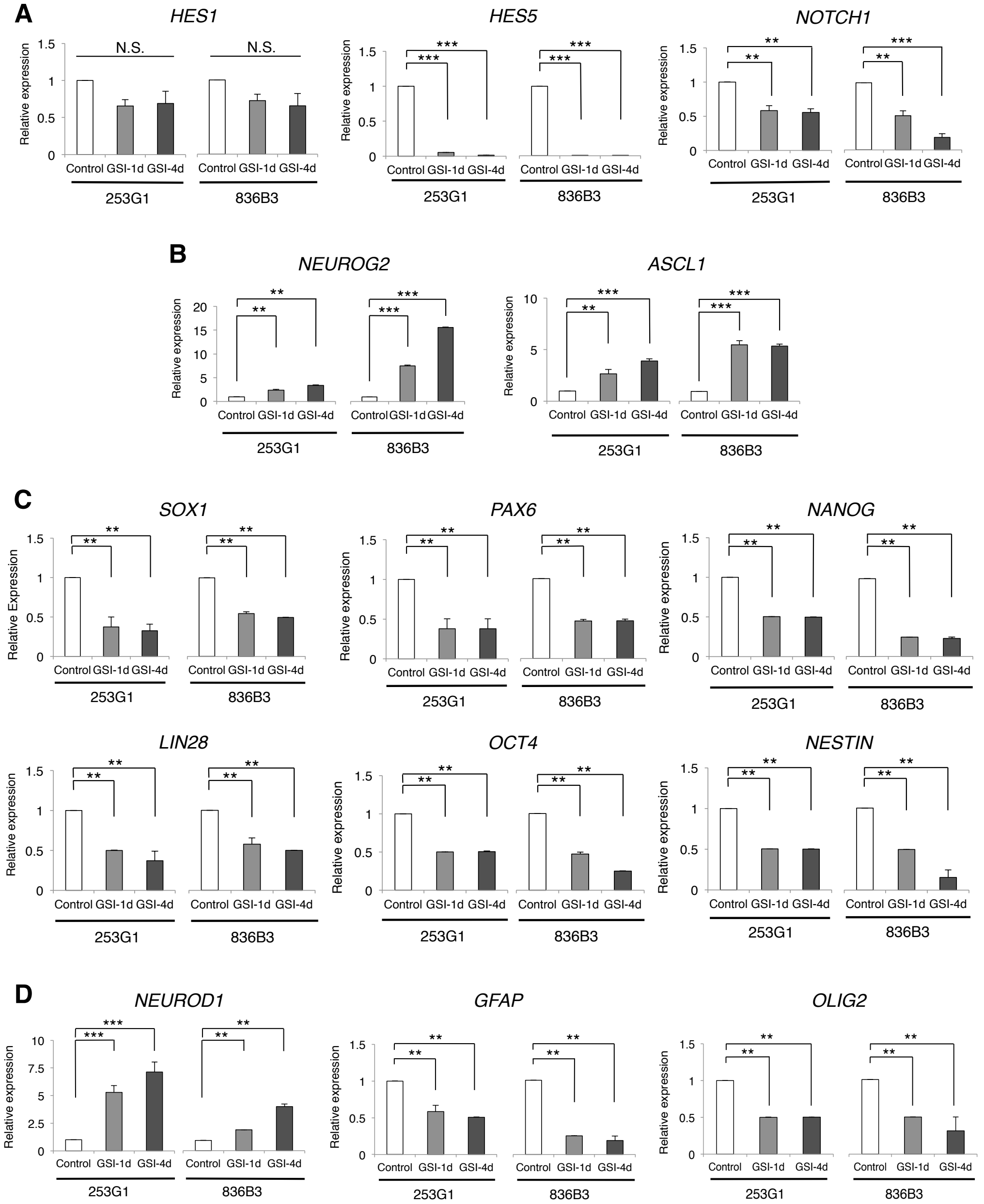

Figure S1. RT-PCR Analyses of hiPSC-NS/PCs Treated with GSI

(A-D) The gene expression of mRNA in the control, GSI-1d and GSI-4d groups were analyzed by RT-PCR for each cell line. Data, normalized to the reference GAPDH levels, were presented as expression levels relative to the control group (equal to 1) (n = 3 independent experiments).

$^{**}p < 0.01$, $^{***}p < 0.001$, N.S. = Non-significant, One-way ANOVA with the Tukey-Kramer test (A-D). The data are presented as the means ± SEM.

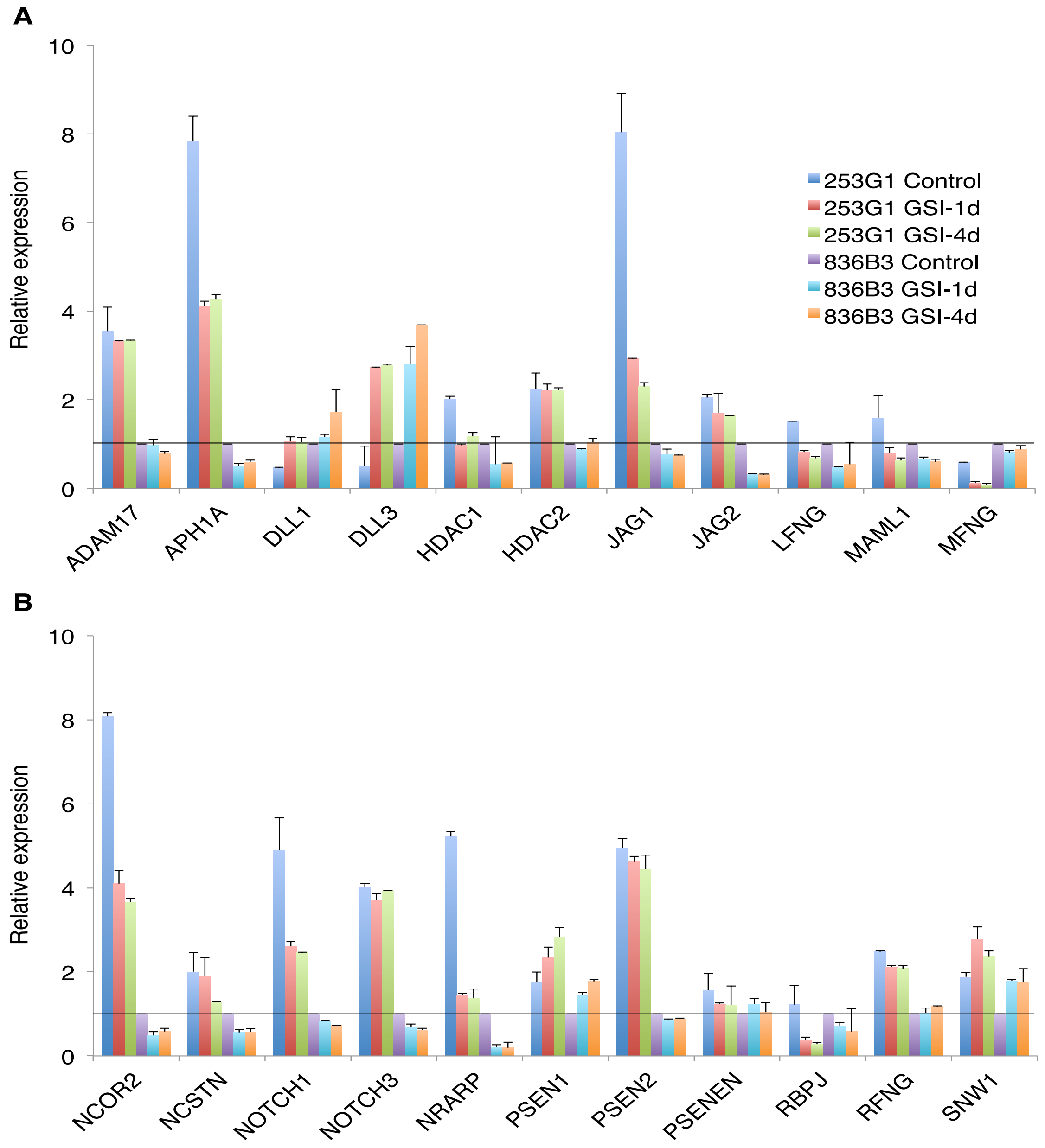

Figure S2. The Gene Expression Analyses of Human Notch Signaling Pathway in the hiPSC-NS/PCs Treated with or without GSI Using RT-PCR

(A, B) Quantitative RT-PCR analyses of known direct and indirect human Notch signal related genes for each group and cell lines. Data, normalized to the reference GAPDH levels, were presented as expression levels relative to the 836B3 hiPSC-NS/PCs control group (equal to 1) (n = 3 independent experiments).

One-way ANOVA with the Tukey-Kramer test (A, B). The data are presented as the means ± SEM.

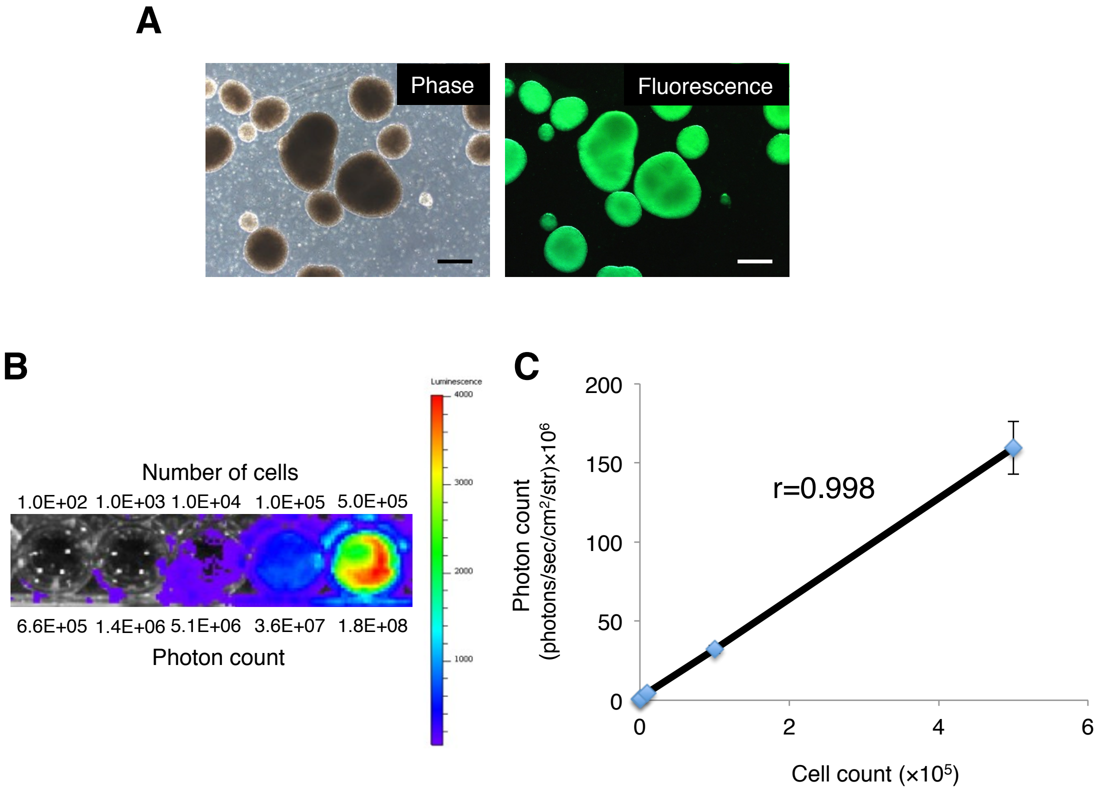

Figure S3. Detection of Bioluminescence and Fluorescence Signals in Lentivirally Transfected 253G1 hiPSC-NS/PCs *in vitro*

(A) Fluorescence image shows neurospheres of 253G1 hiPSC-NS/PCs expressing the fluorescent protein cp156-Venus, which is originally modified from GFP.

(B) Bioluminescence imaging was used to detect bioluminescence signals in various numbers of 253G1 hiPSC-NS/PCs (0, 1×10^2, 1×10^3, 1×10^4, 1×10^5, and 5×10^5 cells per well).

(C) BLI significantly find out a direct linear correlation between cell numbers and photon counts in vitro (n = 5 independent experiments).

The data are presented as the means ± SEM. Scale bars, 200 µm (A).

BLI; bioluminescence imaging

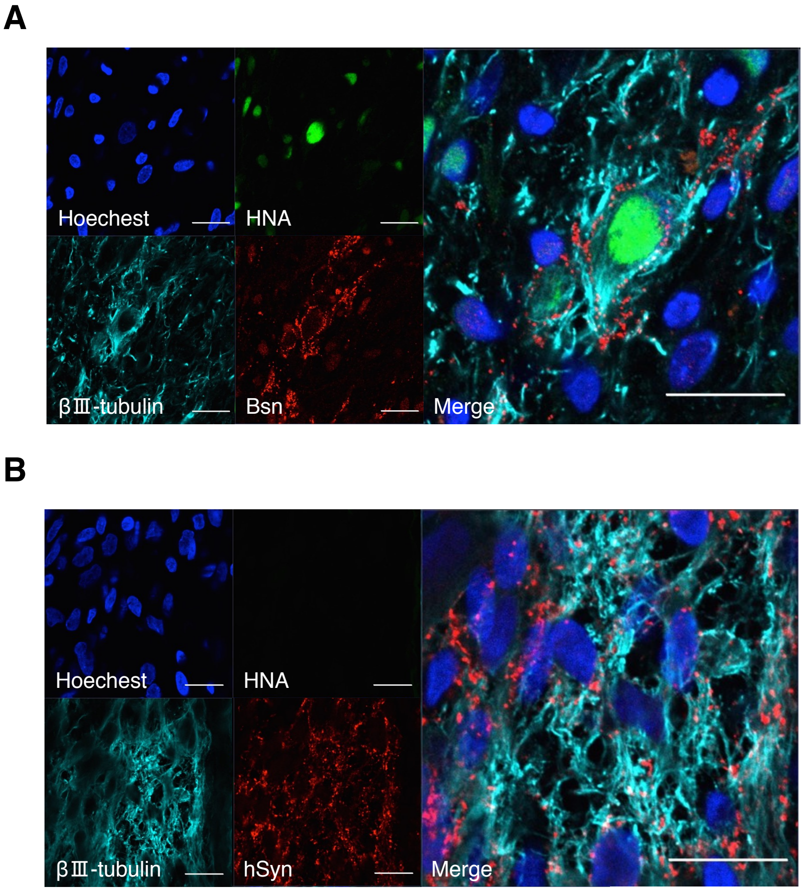

Figure S4. Transplanted hiPSC-NS/PCs Contribute to Synapse Formation between Transplanted Cell-Derived Neurons and Host Mouse Neurons

(A) Representative images of immunohistochemistry show the staining for HNA, βⅢ tubulin, and the mouse presynaptic marker Bsn. βⅢ tubulin^{+}/HNA^{+} transplanted cell-derived neurons were observed in contact with Bsn^{+} cells.

(B) Representative images of immunohistochemistry show the staining for HNA, βⅢ tubulin, and the human-specific presynaptic marker hSyn. hSyn^{+} boutons were apposed to βⅢ tubulin^{+}/HNA^{-} host mouse neurons.

Scale bars, 20μm (A and B).

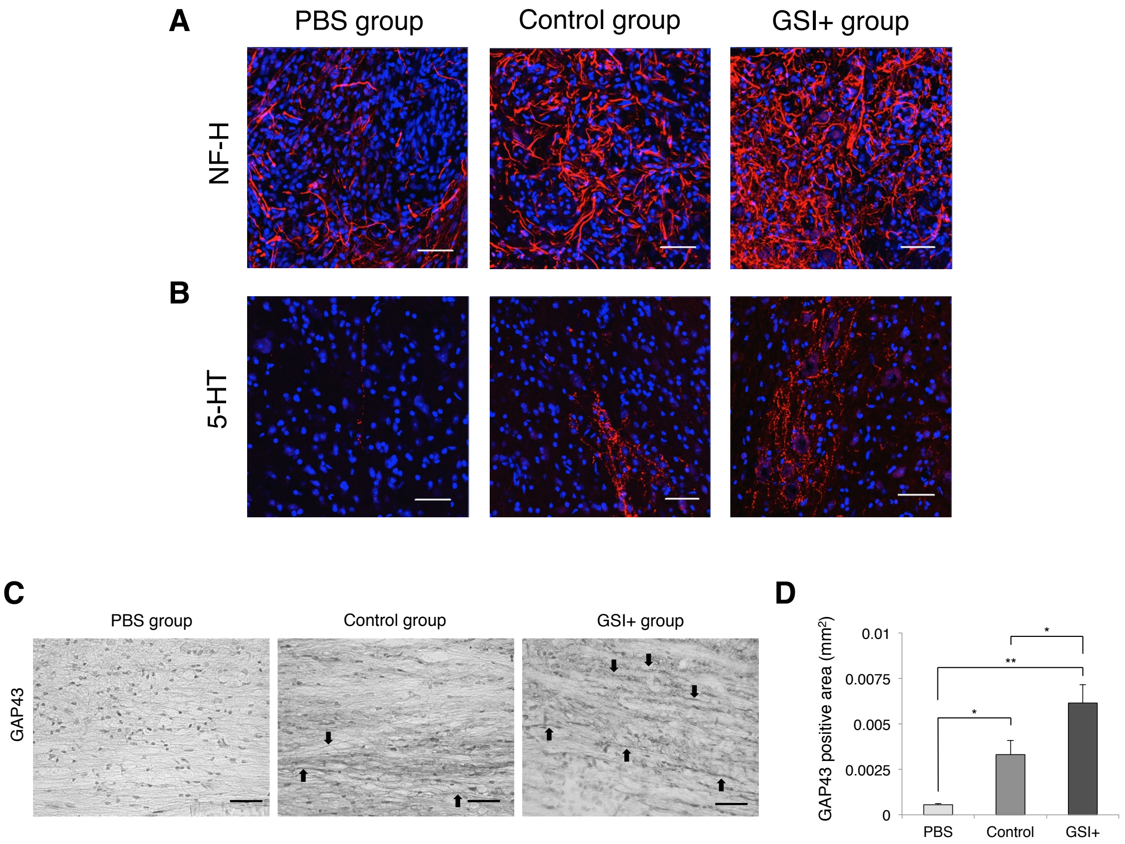

Figure S5. Transplanted hiPSC-NS/PCs with GSI Treatment Enhanced Axonal Regrowth after SCI.

(A) Representative images of the immunohistochemical staining for NF-H at the lesion epicenter, and (B) 5-HT at the lumbar intumescence, show the results for each group.

(C) Representative images of midsagittal sections stained for GAP43 in the ventral region 1 mm caudal to the lesion epicenter. Black arrows indicate the $GAP43^{+}$ fibers

(D) Quantitative analyses of the GAP43+ area in midsagittal sections.

$^{*}p < 0.05$, $^{**}p < 0.01$ according to a one-way ANOVA with the Tukey-Kramer test (D). The data are presented as the means ± SEM. Scale bars, 50μm (A-C).

Table S1. Gene Ontology analysis of human genes upregulated and downregulated decreased in 253G1 hiPS-NS/PCs after pretreated with GSI-1d and GSI-4d

GO:Accession	GO Term	Corrected p-value
GO:0030182	neuron differentiation	1.38E-17
GO:0048666	neuron development	7.31E-09
GO:0007409	axonogenesis	8.90E-09
GO:0048812	neuron projection morphogenesis	9.17E-09
GO:0031175	neuron projection development	2.22E-07
GO:0007411	axon guidance	6.05E-07
GO:0051726	regulation of cell cycle	6.58E-03
GO:0021700	developmental maturation	6.80E-03
GO:0043066	negative regulation of apoptosis	1.17E-02
GO:0043069	negative regulation of programmed cell death	1.31E-02
GO:0060548	negative regulation of cell death	1.35E-02
GO:0010717	regulation of epithelial to mesenchymal transition	1.56E-02
GO:0042981	regulation of apoptosis	1.85E-02
GO:0010941	regulation of cell death	2.17E-02
GO:0008219	cell death	4.45E-02

Table S2. Gene Ontology analysis of human genes upregulated and downregulated decreased in 836B3 hiPS-NS/PCs after pretreatment with GSI-1d and GSI-4d

GO:Accession	GO Term	Corrected p-value
GO:0030182	neuron differentiation	3.65E-07
GO:0048666	neuron development	1.31E-04
GO:0048663	neuron fate commitment	2.08E-04
GO:0021700	developmental maturation	7.15E-04
GO:0048469	cell maturation	1.89E-03
GO:0045597	positive regulation of cell differentiation	5.47E-03
GO:0051726	regulation of cell cycle	8.66E-03
GO:0001764	neuron migration	1.02E-02
GO:0010720	positive regulation of cell development	1.25E-02
GO:0021515	cell differentiation in spinal cord	1.31E-02
GO:0021954	central nervous system neuron development	2.11E-02
GO:0021510	spinal cord development	3.01E-02
GO:0021953	central nervous system neuron differentiation	3.20E-02
GO:0031175	neuron projection development	3.42E-02
GO:0007409	axonogenesis	4.69E-02

Supplemental Experimental Procedures

Cell Culture, NS/PCs Derived from hiPSC-NS/PCs Formation Assay, Neuronal Differentiation Analyses and Lentiviral Transduction

Three human induced pluripotent stem cells (hiPSCs) lines; clone 253G1 (Nakagawa et al., 2008) generated following retroviral transfection of 3-factors (Oct3/4, Sox2 and Klf4) and clone 836B3 (Maekawa et al., 2011; Okita et al., 2011) reprogrammed with episomal plasmid vectors containing 6-factors (Oct3/4, Sox2, Klf4, L-Myc, LIN28 and Glis1), a potentially tumor-like overgrowth, and normal clone 201B7 (Miura et al., 2009) generated following retroviral transfection of 4-factors (Oct3/4, Sox2, Klf4 and c-myc) were cultured for 12 days on adhesion culture with mouse embryonic fibroblast (MEF), and formed into embryo bodies in floating culture for 30 days. Aggregate cells were differentiated into NS/PCs derived from hiPSC-NS/PC formation using various factors during each day of the incubation period. In the analyses of neuronal differentiation, hiPSC-NS/PCs were plated onto poly-L-ornithine/fibronectin-coated 48-well chamber slides (Costar 3548; Corning, NY, USA) at a density of 1×10^5 cells/ml and cultured in medium without growth factors at 37 °C in 5% CO_2 and 95% air for 14 days. Differentiated cells were fixed with 4% paraformaldehyde (PFA) in 0.1 M phosphate buffered saline (PBS) and stained with the following primary antibodies for immunocytochemistry: anti-Ki67 (rabbit IgG, 1:500, Leica Biosystems, Wetzlar, Germany), anti-human-specific Nestin protein (mouse IgG1, 1:500, Merck Millipore, Billerica, Massachusetts, USA), anti-βⅢ-tubulin (mouse IgG2b, 1:500, Sigma-Aldrich, St. Louis, MO, USA), anti-glial fibrillary acidic protein (GFAP, rabbit IgG, 1:200, Dako, Carpinteria, CA, USA), and anti-Cyclicnucleotide phosphodiesterase (CNPase, mouse IgG1, 1:4000, Sigma-Aldrich, St. Louis, MO, USA). Nuclei were stained with Hoechst 33258 (10 μg/ml, Sigma-Aldrich, St. Louis, MO, USA). All *in vitro* images were obtained using a confocal laser scanning microscopy (LSM 700; Carl Zeiss, Jena, Germany / In Cell Analyzer 6000; GE health care UK Ltd, Little Chalfont, Buckinghamshire, UK).

Flow Cytometric Analyses

EdU was added to the culture medium at a final concentration of 10 μM and mixed well for one hour at 37 ℃. Subsequently, cells were washed with 1% Bovine serum albumin (BSA) in PBS, removed the supernatant, fixed for 15 minutes, incubated with Click-iT Plus reaction cocktail (anti-EdU antibodies included) for 30 minutes, and stained FxCycle™ Violet (Life technologies, Carlsbad, CA, USA). Flow cytometry and data

analyses were performed on a fluorescence-activated cell sorting (FACS) Calibur instrument (BD Biosciences, San Jose, CA, USA).

Micro Electrode Array

hiPSC-NS/PCs were dissociated into single cells and also plated in culture medium on the human laminin-coated MEA plates (*n* = 3, 12 well plate, Axion Biosystems, Atlanta, GA, USA). The data were acquired using a sampling rate of 12.5 kHz and filtered using a 200–3000 Hz Butterworth band-pass filter. A detection threshold was set to +6.0×SD of the baseline electrode noise. The spike count files generated from recordings were used to calculate the number of active electrodes, which were defined as an electrode having an average of more than five spikes/min in each well. The first three minutes of each data file was removed in order to allow the activity to stabilize in the Maestro and 10-15 min of activity was recorded. MEA data analyses were performed by NeuroExplorer (Nex Technologies, Madison, AL, USA) software, which normalized the baseline neuronal activity and plotted as a graph for each condition.

DNA Microarray Analyses

Total RNA was extracted from the hiPSC-NS/PCs using an RNeasy Micro Kit (Qiagen Inc., Hilden, Germany) according to the manufacturer's instructions. For the DNA microarray analyses, the RNA quality was assessed using a 2100 Bioanalyzer (Agilent Technologies Inc., Santa Clara, CA, USA), and 100 ng of total RNA was reverse transcribed, biotin labeled, and hybridized to a Human HT-12v4.0 Expression BeadChip (Illumina Inc., San Diego, CA, USA). The array was then washed and stained in a Fluidics Station 450, according to the manufacturer's instructions. The microarrays were scanned using an iScan system (Illumina Inc., San Diego, CA, USA), and the raw image files were converted to normalized signal intensity values using the GenomStudio algorithm (Illumina Inc., San Diego, CA, USA). The results were normalized and narrowed down by the cut-off values for the expression levels (>50) and by the fold changes (>2.0, versus the signal of the 'Control group'). GO analyses were performed using gene lists from the overlapping area in the Venn diagram of each hiPSC-NS/PC. P values were calculated using Fisher's exact test.

RT-PCR

Total RNA was extracted from hiPSC-NS/PCs by using RNeasy Micro Kit (Qiagen Inc., Hilgen, Germany), and reverse transcribed with the ReverTra Ace qPCR RT master mix (TOYOBO co., Ltd. Life science

department, Osaka, Japan). Quantitative polymerase chain reactions (QT-PCR) were performed using Step One Plus^{TM} (Applied Biosystems, Foster city, CA, USA), according to the manufacturer's instructions. The expression levels of each gene were normalized to that of GAPDH using the ΔΔCT method. We chose primers used for the reactions from TaqMan gene expression assays and TaqMan gene expression 96-well fast plates (Life technologies, Carlsbad, CA, USA).

Bioluminescence Imaging

A Xenogen-IVIS spectrum cooled, charge coupled device (CCD) optical macroscopic imaging system (Caliper Life-Science, Hopkinton, MA, USA) was used for BLI to evaluate the survival of the hiPSC-NS/PCs grafted as described in the literature (Itakura et al., 2014; Okada et al., 2005; Takahashi et al., 2011). The signal intensity of hiPSC-NS/PCs was assessed *in vitro* using cells plated at various cell numbers (approximate range 1×10^2 to 5×10^5 cells/well), and BLIs were performed immediately after adding D-luciferin (150 μg/ml; Promega, Madison, WI, USA, n=3). The integration time was fixed at five minutes for each image. Imaging was performed *in vivo* five minutes after via i.p. injection of D-luciferin (0.3 mg/g body weight) with the field-of-view set at 13.2 cm, as the photon count was most stable during this period. The intensity peaked between 10 and 30 minutes. All images were measured with Living Image software, and the optical signal intensity was expressed as photon count in units of photons/sec/cm^2/str. Each result was displayed as a pseudo-colored photon count image superimposed on a gray-scale anatomic image. A region of interest was defined in the area cell-implanted, and all values at the same region of interest were elucidated to quantify the light measured. The survival and growth of cells transplanted in the mouse spinal cord was measured by BLI for IVIS system every week until 89 days after transplantation.

SCI Animal Model and hiPSC-NS/PCs Transplantation

Adult female NOD-SCID mice (8-weeks-old, 20-22 g; Clea, Tokyo, Japan) were anesthetized with intra-peritoneal (i.p.) injection of ketamine (100 mg/kg) and xylazine (10 mg/kg). After laminectomy at the level of 10th thoracic spinal vertebra, the dorsal surface of the dura mater was exposed, and contusive SCI was induced using an IH impactor (a force defined impact (60 kdyn) with a stainless steel-tipped impactor; Precision Systems and Instrumentation, Lexington, KY, USA) as described previously (Scheff et al., 2003). Donor cells were prepared *in vitro* from 253G1 and 201B7 hiPSC-NS/PCs, which were cultured with GSI for one day in 'GSI+ group' and without GSI in 'control group' before cell transplantation. Nine days after SCI,

hiPSC-NS/PCs pretreating with or without GSI (5×10^5 cells/2 μl) were transplanted into the lesion epicenter of each mouse ('control group' and 'GSI+ group', n=10 each) with a glass micropipette at a rate of 1 μl/min using a Hamilton syringe (25 μl) and a stereotaxic micro injector (KDS 310; Muromachi Kikai Co. Ltd., Tokyo, Japan). An equal volume of PBS was injected instead of 'PBS group' mice.

Histological analyses

Animals were anesthetized and transcardially euthanized with 0.1 M PBS containing 4% PFA at 89 days after transplantation. Spinal cords were removed, post-fixed overnight in 4% PFA, soaked overnight in 10% sucrose, followed by 30% sucrose, embedded in Optimal Cutting Temperature (O.C.T) compound (Sakura Finetechnical Co., Ltd., Tokyo, Japan), frozen, and sectioned in the sagittal plane at 16 μm thickness on a cryostat (CM3050S, Leica Microsystems, Wetzlar, Germany). Spinal cord sections were histologically evaluated by staining with HE and immunohistochemistry (IHC). Especially, tissue sections were applied and stained with the following primary antibodies for IHC: anti-pan-ELAVL (Hu) (human IgG, 1:1000, a gift from Dr. Robert Darnell, The Rockefeller University, New York, NY, USA), anti-GFAP (rabbit IgG, 1:200, Dako, Carpinteria, CA, USA, Z0334), anti-APC CC-1 (mouse IgG, 1:300; Abcam, Cambridge, UK, ab16794), anti-human-specific Nestin protein (rabbit IgG, 1:300; described previously (Kanemura et al., 2002; Nakamura et al., 2003)), anti-Ki67 (rabbit IgG, 1:200; Leica Biosystems, Wetzlar, Germany), anti-human nuclear antigen (anti-HNA, mouse IgG, 1:200; Chemicon, Temecula, CA, USA, MAB1281), anti-Notch1 (rabbit IgG, 1:100; Abcam, Cambridge, UK, ab65297), anti-activated Notch1 (rabbit IgG, 1:100; Abcam, Cambridge, UK, ab8925), anti-Snail (rabbit IgG, 1:100; Abcam, Cambridge, UK, ab180714), anti-βⅢ-tubulin (Tuj1; mouse IgG, 1:300; Sigma-Aldrich, St. Louis, MO, USA, T8660), anti-Bsn (mouse IgG, 1:200; Stressgen, ADI-VAM-PS003), anti-hSyn (Mouse IgG, 1:200; Chemicon, Temecula, CA, USA, MAB332), anti-neurofilament 200 kDa (NF-H, mouse IgG, 1:200; Merck Millipore, Billerica, Massachusetts, USA, MAB5266), anti-5-hydroxytrypamine (5HT, goat IgG, 1:200; Immunostar), anti-growth associated protein 43 (GAP43, mouse IgG, 1:1000; Chemicon, Temecula, CA, USA). For immunohistochemistry with anti-GAP43, we used a biotinylated secondary antibody (Jackson Immunoresearch Laboratory, West Grove, PA, USA), after exposure to 0.3% H_2O_2 for 60 min at room temperature to inactivate the endogenous peroxidase. The signals were then en- hanced with the Vectastain ABC kit (Vector Laboratories, Burlingame, CA, USA). Nuclei were stained with Hoechst 33258 (10 μg/ml, Sigma-Aldrich, St. Louis, MO, USA). Samples were elucidated on a fluorescence microscope (BZ 9000; Keyence Co., Osaka, Japan) or a confocal laser-scanning

microscope (LSM 700, Carl Zeiss, Jena, Germany). The numbers of marker-positive cells, such as HNA-, Ki67-, Nestin-, pan-ELAVL (Hu)-, GFAP-, APC-, Notch1-, Activated Notch1-, Snail- positive cells, were counted in each section (n = five per group).

Behavioral Analyses

Two investigators blinded to identify the experimental mice performed this assessment. Motor coordination was evaluated using a rotating rod apparatus (Rota-rod, Muromachikikai Co., Ltd.), which consisted of a plastic rod (three cm diameter, eight cm length) with a gridded surface flanked by two large discs (40 cm diameter). Each mouse was placed on the rod while it rotated at 20 rpm for two minutes sessions at 89 days after transplantation. Five trials were conducted, and the maximum number of seconds the mouse stayed on the rod was recorded. Gait analyses were performed using the DigiGait system (Mouse Specifics, Quincy, MA, USA, n=five per group). Each mouse demonstrated weight-supported hindlimb stepping at 89 days after transplantation. Stride length was determined on a treadmill set to a speed of seven cm/s.

Supplemental References

Maekawa, M., Yamaguchi, K., Nakamura, T., Shibukawa, R., Kodanaka, I., Ichisaka, T., Kawamura, Y., Mochizuki, H., Goshima, N., and Yamanaka, S. (2011). Direct reprogramming of somatic cells is promoted by maternal transcription factor Glis1. Nature *474*, 225-229

Nakagawa, M., Koyanagi, M., Tanabe, K., Takahashi, K., Ichisaka, T., Aoi, T., Okita, K., Mochiduki, Y., Takizawa, N., and Yamanaka, S. (2008). Generation of induced pluripotent stem cells without Myc from mouse and human fibroblasts. Nat. Biotechnol. *26(1)*, 101-106

Okita, K., Matsumura, Y., Sato, Y., Okada, A., Morizane, A., Okamoto, S., Hong, H., Nakagawa, M., Tanabe, K., Tezuka, K., et al. (2011). A more efficient method to generate integration-free human iPS cells. Nature Methods *8*, 409-412
